# MAST4 regulates stem cell maintenance with DLX3 for epithelial development and amelogenesis

**DOI:** 10.1038/s12276-024-01264-5

**Published:** 2024-07-01

**Authors:** Dong-Joon Lee, Pyunggang Kim, Hyun-Yi Kim, Jinah Park, Seung-Jun Lee, Haein An, Jin Sun Heo, Min-Jung Lee, Hayato Ohshima, Seiya Mizuno, Satoru Takahashi, Han-Sung Jung, Seong-Jin Kim

**Affiliations:** 1 Division in Anatomy and Developmental Biology, Department of Oral Biology, Taste Research Center, Oral Science Research Center, BK21 FOUR Project, Yonsei University College of Dentistry, Seoul, 03722 Korea; 2 Department of Oral Histology, Dankook University College of Dentistry, Cheonan, 31116 Korea; 3 Institute of Tissue Regeneration Engineering (ITREN), Dankook University, Cheonan, 31116 Korea; 4 GILO Institute, GILO Foundation, Seoul, 06668 Korea; 5 NGeneS Inc., Ansan-si, Gyeonggi-do 15495 Korea; 6 Division of Anatomy and Cell Biology of the Hard Tissue, Department of Tissue Regeneration and Reconstruction, Niigata University Graduate School of Medical and Dental Sciences, Niigata, 951-8514 Japan; 7 Laboratory Animal Resource Center, University of Tsukuba, Tsukuba, Ibaraki 305-8575 Japan; 8 Medpacto Inc., Seoul, 06668 Korea

**Keywords:** Self-renewal, Transcriptional regulatory elements, Phosphorylation

## Abstract

The asymmetric division of stem cells permits the maintenance of the cell population and differentiation for harmonious progress. Developing mouse incisors allows inspection of the role of the stem cell niche to provide specific insights into essential developmental phases. Microtubule-associated serine/threonine kinase family member 4 (Mast4) knockout (KO) mice showed abnormal incisor development with low hardness, as the size of the apical bud was decreased and preameloblasts were shifted to the apical side, resulting in amelogenesis imperfecta. In addition, Mast4 KO incisors showed abnormal enamel maturation, and stem cell maintenance was inhibited as amelogenesis was accelerated with *Wnt* signal downregulation. Distal-Less Homeobox 3 (DLX3), a critical factor in tooth amelogenesis, is considered to be responsible for the development of amelogenesis imperfecta in humans. MAST4 directly binds to DLX3 and induces phosphorylation at three residues within the nuclear localization site (NLS) that promotes the nuclear translocation of DLX3. MAST4-mediated phosphorylation of DLX3 ultimately controls the transcription of DLX3 target genes, which are carbonic anhydrase and ion transporter genes involved in the pH regulation process during ameloblast maturation. Taken together, our data reveal a novel role for MAST4 as a critical regulator of the entire amelogenesis process through its control of *Wnt* signaling and DLX3 transcriptional activity.

## Stem cell niche insights: MAST4 regulates dental stem cells and amelogenesis

The research examines the function of MAST4, a protein, in tooth growth, particularly in creating enamel (the hard, outer layer of the tooth). Scientists discovered that mice without MAST4 had unusual enamel development in their front teeth, resulting in weaker teeth. The research showed that MAST4 is vital for preserving stem cells (cells that can develop into many different cell types) and controlling their transformation into ameloblasts (cells that create enamel). Without MAST4, this process was disrupted, causing early enamel release and incorrect maturation. The scientists also discovered that MAST4 controls the function of another protein, DLX3, necessary for enamel maturation. This research offers a new understanding of the molecular processes involved in tooth growth and could be significant for understanding and treating dental issues.

This summary was initially drafted using artificial intelligence, then revised and fact-checked by the author.

## Introduction

During tooth development, ameloblasts derived from dental epithelial cells and odontoblasts derived from cranial neural crest mesenchymal cells are responsible for the formation of enamel and dentin, respectively. In particular, rodent incisors are an attractive model for studying the molecular and cellular events involved in stem cell maintenance and differentiation^1^. Recent advances in research techniques have revealed the single-cell transcriptomes of all cell populations that form incisors^2,3^, and once again, the incisor is in the spotlight as a model organ component for epithelial stem cell research. The early process of mouse incisor development resembles molar development, including initiation at embryonic day 11 (E11). However, only the labial epithelium shows asymmetrical development of differentiation into ameloblasts^4^. Mouse incisors can grow throughout the animal’s lifespan, and adult mouse incisors contain cells at all stages of tooth development in the epithelial compartment, from epithelial stem cells in the labial cervical loop (also called the apical bud) to fully differentiated ameloblasts. Stem cell progeny leave the niche of the apical bud, enter a transit-amplifying (TA) zone, and proliferate^5^. Then, these cells differentiate, secrete matrix components, and finally undergo apoptosis, simultaneously gradually advancing toward the distal tip of the incisor. Due to this spatial linearity, akin to a conveyor belt, tissue renewal occurs in an easily observed proximodistal fashion; thus, cells at increasingly advanced stages of maturation are found at progressively more distal locations^3^. This epithelial differentiation to ameloblasts eventually leads to amelogenesis, and the three major stages of amelogenesis are the secretory, transition, and maturation stages. Moreover, ameloblast cell‒cell attachment, detachment, and movement are regulated so that the rodent characteristic decussating enamel rod pattern can form during the secretory stage of amelogenesis^6,7^.

Preameloblasts differentiate into secretory ameloblasts that deposit an extracellular matrix consisting of proteins such as amelogenin, ameloblastin, enamelin, tuftelin, and MMP20^8–11^, and mineralization is then initiated. A shift from matrix deposition to resorption occurs at the transitional and maturation stages, as indicated by the predominant expression of *Mmp20*, *Klk4*^11^, *Amelotin*^12,13^, and *Odam*^14^. Ablation of *Mmp20* in mice causes enamel to become thin and brittle, and flake off the underlying dentin^7^. KLK4 is a serine protease expressed during enamel maturation, and proteolytic processing of the enamel matrix by KLK4 is critical for normal enamel formation. Two proteases are secreted into the enamel matrix of developing teeth. The early protease is MMP20, and the late protease is KLK4. Mutations in both *Mmp20* and *Klk4* cause the autosomal recessive condition amelogenesis imperfecta (AI), which is a genetic disorder characterized by morphological and functional defects in tooth enamel formation and featuring soft, porous enamel containing residual proteins^15^.

Signaling networks that communicate and interact with FGF3 and WNT expressed in pulp mesenchymal cells are believed to regulate the maintenance and differentiation of epithelial stem cells in the cervical loop^16,17^. The Pitx2-Sox2-Lef1 transcriptional axis is also reported to regulate dental epithelial stem cell homeostasis^18,19^. *Wnt* signaling plays an important role in regulating cell proliferation, differentiation, and polarity^20,21^. The canonical *Wnt* pathway mediates signaling by regulating the intracellular level and subcellular localization of β-catenin^22^. An in vitro study of dental pulp cells revealed that *Wnt* signaling is downregulated by Distal-Less Homeobox 3 (DLX3) through the regulation of DKK1 expression^22^. However, a recent study reported that *Wnt* signaling could also be upregulated by DLX3 through the suppression of DKK4^23^. In addition, in hair follicles, *Dlx3* acts downstream of *Wnt*^24^. As such, DLX3 and *Wnt* signaling have a complex relationship.

Mutations in several genes, including *Amelogenin* (*Amelx*), *Ameloblastin (Ambn)*, *Enamelin (Enam)*, *Matrix metalloproteinase-20* (*Mmp20*), *Kallikrein-4* (*Klk4*), *Dlx3*, *WD Repeat domain-72* (*Wdr72*), *Family with Sequence Similarity 83 Member H* (*Fam83h*), and *Fam20a*^9,25–28^, are responsible for AI in humans. Among the corresponding encoded proteins, DLX3 is a transcription factor that promotes the expression of enamel matrix proteins during amelogenesis^29^. In addition, a study using mice with ameloblast-specific conditional knockout (KO) of *Dlx3* showed that DLX3-mediated regulation of target genes related to pH regulation for enamel maturation was more important than that of genes encoding enamel matrix proteins^30^, suggesting a complex mechanism underlying the role of DLX3 as a transcription factor during tooth development. Previous studies revealed that the DNA binding activity of DLX3 is regulated by the phosphorylation of serine 138 by protein kinase C in keratinocytes and that DLX3 stability is regulated by the phosphorylation of serine 10 by protein kinase A during osteoblast differentiation^31,32^. However, studies on the phosphorylation of DLX3 during tooth development have not yet been conducted. Microtubule-associated serine/threonine kinase family member 4 (*Mast4*) is a human protein kinase that was identified in 2006^33^. To date, very little research has been performed on the developmental role of MAST4, and most related studies have been limited to its role in the brain and mental disorders^34–36^. Recently, Mast4 has been shown to play pivotal roles in the bone/cartilage differentiation of mesenchymal stem cells and in spermatogonial stem cell renewal^37,38^. Phosphorylation of DLX3 by MAST4 has also not been studied.

In this study, we demonstrated by physicochemical and histological methods that the incisor teeth of *Mast4* KO mice exhibited significantly different incisor phenotypes. Through RNA sequencing analyses of the isolated secretory ameloblasts and apical buds of *Mast4* KO mice, we confirmed an increases in the expression of amelogenesis-related genes and decreases in the expression of *Wnt* signaling pathway-related genes. In addition, we found that the regulation of the nuclear localization of DLX3 by MAST4 seems to influence the physiological condition of the incisor enamel matrix. Here, we report a unique role of MAST4, which is expressed in the ameloblast layer in postnatal growing mouse incisors. These studies highlight the importance of MAST4 as a key regulator of mouse incisor amelogenesis and stem cell maintenance in the apical bud.

## Materials and methods

### Animals

All animal experiments were approved by the Yonsei University Health System Institutional Animal Care and Use Committee (YUHS-IACUC) and were conducted in accordance with the Guide for the Care and Use of Laboratory Animals (National Research Council, USA). The animal study plan for these experiments (2017-0206) was reviewed and approved by the above committee.

All the mice were housed in a temperature‐controlled room (22 °C) under artificial illumination (lights on from 05:00 to 17:00) at 55% relative humidity, and they had ad libitum access to food and water. All the surgical procedures were performed under deep anesthesia.

To generate *Mast4* KO mice via CRISPR/Cas9-mediated gene editing, we targeted exon 1 of *Mast4* (RefSeq Accession Number: 175171) with the guide RNA sequence 5’- GGAAACTCTGTCGGAG GAAG-3’ (exon 1). We then inserted each sequence into the pX330 plasmid, which carried both the guide RNA and Cas9 expression units, obtained from Dr. Feng Zhang (Addgene plasmid 42230)^39^. We named these vectors pX330-Mast4-E1 and pX330-Mast4-E15. A schematic of the targeted exon 1 and the translated peptide is shown in Supplementary Fig. 1.

Pregnant mare serum gonadotropin (five units) and human chorionic gonadotropin (five units) were intraperitoneally injected into female C57BL/6J mice (Charles River Laboratories, Kanagawa, Japan) at 48-h intervals, and these mice were subsequently mated with male C57BL/6J mice. pX330-Mast4-E1 and pX330-Mast4-E15 (circular, 5 ng/μl each) were microinjected together into 231 zygotes collected from the oviducts of mated female mice. The 225 surviving injected zygotes were transferred into the oviducts of pseudopregnant ICR female mice, and 47 neonates were obtained. Genomic DNA was collected from the tails of 31 surviving founder mice.

To confirm the indel mutation induced by CRISPR/Cas9, we amplified the genomic region, including the target sites, by PCR with primers for the exon 1 target (Supplementary Table 1). The PCR products were sequenced using the BigDye Terminator v3.1 Cycle Sequencing Kit (Thermo Fisher Scientific) and the Mast4-1 genotype F primer. In male founder #38, we detected indel mutations in both exon 1 and exon 15 without random pX330 integration. To identify the indel sequence and determine whether the indel mutations in exon 1 occurred on the same chromosome (*cis* manner), founder #38 was mated with a wild-type female, and the indel mutations in F1 were sequenced. We obtained 17 F1 newborns, 12 of which carried a 71 bp deletion (chr13:103,333,981-103,334,051: GRCm38/mm10) in exon 1 in a cis manner.

### Vickers hardness test

Erupted portions of incisors from 6-week-old WT and *Mast4* KO littermate mice were washed and dehydrated through an alcohol gradient. The incisors were embedded in the sagittal plane in a hard-formulation epoxy embedding medium (EpoFix, EMS, Hatfield, PA, USA). The samples were ground and polished with an EcoMet 30 grinder polisher (Buehler, IL, USA) with 1500 grit sandpaper at 400 rpm and 0.25 μm. The enamel microhardness of the polished samples was measured using an MMT-X testing instrument (Matsuzawa, Akita, Japan). Testing was performed with a load of 25 g for 5 s using a Vickers tip. Five indentations per sample were performed on eight incisors (four maxillary and four mandibular) per group, and each indentation was measured.

### Cell culture

The mHat9d cell line was obtained from Professor Harada’s laboratory (Iwate Medical University, Japan). mHat9d is a dental epithelial stem cell line derived from the apical bud epithelium of a mouse incisor. The cells were cultured in a 1:1 mixture of Dulbecco’s modified Eagle’s medium and Ham’s F-12 medium (DMEM/F-12; #11320-033, Life Technologies, USA) containing B-27 supplement (#17504-044, Life Technologies, USA), Fibroblast Growth Factor-basic (bFGF; 25 ng/mL, #100-18B, PeproTech, Inc., USA), and Epidermal Growth Factor (EGF; 100 ng/mL, AF-100-15, PeproTech, Inc., USA) at 37 °C in a humidified atmosphere with 5% carbon dioxide (CO_2_). HEK293T cells were grown in DMEM (WELGENE, Korea) supplemented with 10% fetal bovine serum (WELGENE) and 1% penicillin‒streptomycin (WELGENE).

To establish *Mast4*-depleted mHat9d cells, the lentiCRISPRv2 vector (#52961, Addgene, USA) was digested with BsmBI and ligated to an annealed oligonucleotide targeting *Mast4* exon 1 (5’-TACCCTGCCGCTGCCGCACC-3’; LentiCRISPRv2-Mast4 Ex1). The vector without the insert was used as a control. To produce the lentiviruses, HEK293T cells were transfected with LentiCRISPRv2-Mast4 Ex1, and the envelope and packaging plasmids (pVSVG and psPAX2) using FuGENE® (E2311, Promega, USA) at 70% confluence. The viral supernatant was harvested 48 h posttransfection, filtered through 0.45-μm filters, and applied to mHat9d cells. Cell clones were selected with puromycin (A11138-03, Life Technologies, USA) at 48 h posttransfection. To establish retrovirus-based Mast4-overexpressing mHat9d cells, both the control LPCX and MAST4 PDZ-LPCX vectors were transfected with pVSVG into GP2-293 cells. The supernatant containing the recombinant retroviruses was collected 36 h after transfection and filtered through a 0.45-μm sterilization filter. Viral transduction and puromycin selection were performed using the same protocol used for the lentiviruses.

### RNA sequencing

Libraries were prepared for 150 bp paired-end sequencing using a TruSeq Stranded mRNA Sample Preparation Kit (Illumina, CA, USA). Specifically, mRNA molecules were purified from 1 μg of total RNA using oligo dT magnetic beads and fragmented. The mRNA fragments were reverse-transcribed into single-stranded cDNAs with random hexamer primers. By applying the generated cDNA as a template for second-strand synthesis, double-stranded cDNA was prepared. After sequential end repair, A-tailing and adapter ligation, cDNA libraries were amplified via PCR (polymerase chain reaction). The quality of these cDNA libraries was evaluated with an Agilent 2100 BioAnalyzer (Agilent, CA, USA), and they were quantified with a KAPA library quantification kit (Kapa Biosystems, MA, USA) according to the manufacturer’s library quantification protocol. Following cluster amplification of the denatured templates, paired-end (2 × 150 bp) sequencing was performed using an Illumina NovaSeq 6000 sequencer (Illumina, CA, USA). Low-quality reads were filtered according to the following criteria: reads containing more than 10% skipped bases (marked as “N”), reads containing more than 40% of bases with a quality score of less than 20 and reads with an average quality score of less than 20. The whole filtering process was performed using in-house scripts. Filtered reads were mapped to the reference genome related to the species using the aligner TopHat^40^. The gene expression level was measured with Cufflinks v2.1.1^41^ using the gene annotation database for the appropriate species. To improve the accuracy of the measurements, the multi-read-correction and frag-bias-correct options were applied. All other options were set to the default values.

### Statistical analysis

The results are presented as the means ± standard deviations (SDs). The data were analyzed using Student’s *t*-test or one-way ANOVA followed by post hoc comparison using Tukey’s test with Prism 7 software (GraphPad, La Jolla, CA). *p* < 0.05 (two-tailed) was considered to indicate statistical significance.

### Methods for molecular biological research

Details of the methods used for the molecular biological experiments, including subcellular fractionation and Western blotting, immunohistochemistry, immunoprecipitation (IP), real-time quantitative PCR (RT‒qPCR), chromatin IP (ChIP), site-directed mutagenesis, and luciferase assays, can be found in the Supplementary Information (SI).

## Results

### Incisor morphology, strength, and enamel composition in *Mast4* KO mice

**Fig. 1:**
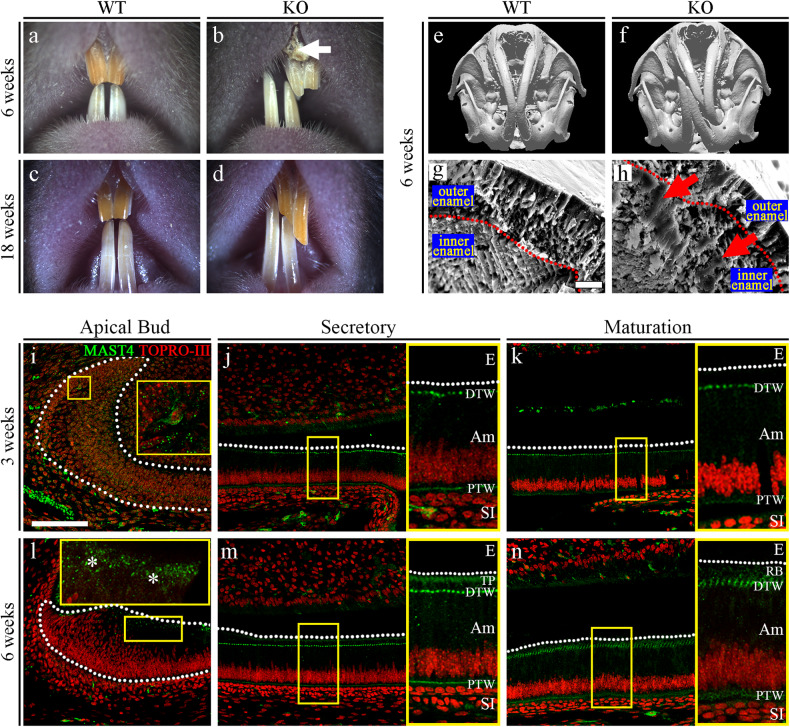
Deletion of *Mast4* leads to an amelogenesis imperfecta phenotype. **a**–**d** Comparison of incisors from 6-week-old and 18-week-old WT and *Mast4* KO mice. Bent maxillary and mandibular incisors were observed in *Mast4* KO mice. The arrow indicates the peeled enamel in a maxillary incisor from a 6-week-old *Mast4* KO mouse. **e**, **f** Micro-CT 3D reconstruction of the head of a *Mast4* KO mouse shows that the incisor is severely curved. **g**, **h** Scanning electron microscopy (SEM) images of incisor enamel dissected planes. The outer enamel of *Mast4* KO incisors was thicker than that of WT incisors. Decussated enamel rods were observed in the inner enamel layer of the WT incisor. The enamel rod arrangement was collapsed in the *Mast4* KO incisor (arrows). **i**–**n** MAST4 localization in the WT incisor. The dotted lines indicate the boundary of the epithelial cell layer. **i**–**k** In incisors from 3-week-old mice, MAST4 was generally weakly expressed at the apical bud, but single cells with high MAST4 expression in the stem cell niche region were also detected. MAST4 was weakly expressed at proximal terminal web complexes (PTW) and moderately distal terminal web complexes (DTW) in secretory and maturation stage ameloblasts. **l** After 6 weeks, MAST4 expression extended to the apical side (asterisks). **m** MAST4 was localized to both sides (proximal and distal) of terminal web complexes and Tomes’ processes (TP) in secretory stage ameloblasts. **n** In the ameloblast maturation stage, MAST4 was observed at the ruffled border (RB) and terminal web complexes (PTW, DTW). E enamel, DTW distal terminal web complex, PTW proximal terminal web complex, TP Tomes’ process, RB ruffled border, Am ameloblast, SI stratum intermedium. Scale bars, **g**, **h**, 10 μm; **l**–**n**, 100 μm.

*Mast4* KO mice with targeted deletion of 71 base pairs in exon 1, which resulted in a premature stop codon resulting from a frameshift mutation, were generated using the CRISPR/Cas9 system (Supplementary Fig. 1). *Mast4* KO mice were grossly similar to their WT littermates after birth in terms of size and shape, particularly those of the cranial skeleton (Supplementary Fig. 2a). However, the maxillary and mandibular incisors of *Mast4* KO mice showed asymmetrical attrition at postnatal week 3 (Supplementary Fig. 2b, arrow). At 6 weeks, the asymmetrical attrition in *Mast4* KO mice became more severe, and bending began to occur (Fig. 1a, b). At 18 weeks, the *Mast4* KO mice were all viable, but they possessed opaque mandibular incisors with chalky surfaces, while the mandibular incisors of WT mice were transparent and glossy (Fig. 1c, d).

We also observed twisting to one side between the maxillary and mandibular incisors in 80 to 90% of the *Mast4* KO mice; the incisors were overgrown, and the enamel of the maxillary incisors was peeled off (Fig. 1b, arrow). Microcomputed tomography (μCT) analysis of the head more clearly revealed the incisor malocclusion in *Mast4* KO mice (Fig. 1e, f). To investigate the developmental patterns of molar dentition, histological analysis was performed at the bell stage (embryonic day 18.5; Supplementary Fig. 3a, b, d, e). Relative to that in the molar tooth germs of WT mice, the ameloblast layer arrangement in the molar tooth germs of *Mast4* KO mice exhibited an irregular shape (Supplementary Fig. 3e, arrowheads). However, there was no difference in morphogenesis, including in cuspal patterning. In addition, there were no differences in molar dentition between WT and *Mast4* KO mice until postnatal week 10 (Supplementary Fig. 3c, f).

Scanning electron microscopy (SEM) analysis was performed to compare the developing enamel between WT and *Mast4* KO mice (Fig. 1g, h and Supplementary Fig. 4). This revealed that the enamel of developing *Mast4* KO mouse incisors contained a collapsed enamel rod arrangement in the inner enamel (Fig. 1h, arrows), specifically in the area adjacent to the outer enamel (Supplementary Fig. 4, arrowheads). Furthermore, electron probe microanalysis (EPMA) was performed to investigate the mineral content of the incisors (Supplementary Fig. 5a, b). We divided the calcified portion of incisors from 6-week-old mice into two parts (the secretory and maturation regions) and proceeded with EPMA. Interestingly, in the maturation region of *Mast4* KO incisors, calcium (Ca), magnesium (Mg), and phosphorous (P) were reduced, whereas there were no significant differences in the secretory region. We then performed a Vickers test to determine the hardness of the incisors of 6-week-old WT and *Mast4* KO mice. The Vickers test revealed that the loss of MAST4 expression significantly reduced enamel hardness (Supplementary Fig. 6). This altered intensity cannot balance the incisor bite force and may lead to a twisted phenotype. Based on these results, the expression pattern of MAST4 was confirmed in various developmental stages of molars and incisors (Supplementary Fig. 7) as well as in three different regions of juvenile (3-week-old) and adult (6-week-old) incisors (Fig. 1i–n). At the E12.5, E14.5, and E16.5 stages, MAST4 was not detected in molars (Supplementary Fig. 7a–c). At postnatal 1 day, while MAST4 was expressed and dispersed in the stellate reticulum (SR), its expression was not detected in ameloblasts (Supplementary Fig. 7d). In the incisors, there was no MAST4 expression at any developmental stage (Supplementary Fig. 7e, f). At 3 weeks, MAST4 was generally weakly expressed, but strong MAST4 expression was observed in a single cell in the stem cell niche of the apical bud (Fig. 1i). However, MAST4 was expressed at proximal and distal terminal web complexes in the secretory and maturation stages (Fig. 1j, k, PTW, DTW). At 6 weeks, MAST4 expression was observed on the apical side (Fig. 1l, asterisks). In particular, MAST4 was detected at Tomes’ processes (Fig. 1m, TP) as well as

terminal web complexes (Fig. 1m, PTW, DTW) in the secretory stage and at the ruffled border of ameloblasts (Fig. 1n, RB) and terminal web complexes (Fig. 1n, PTW, DTW) in the maturation stage. These results suggest that the weakness of the incisors of *Mast4* KO mice compared to those of WT mice may be caused by dysregulation during amelogenesis, not during development.

### Amelogenesis dysregulation in the incisors of *Mast4* KO mice

**Fig. 2:**
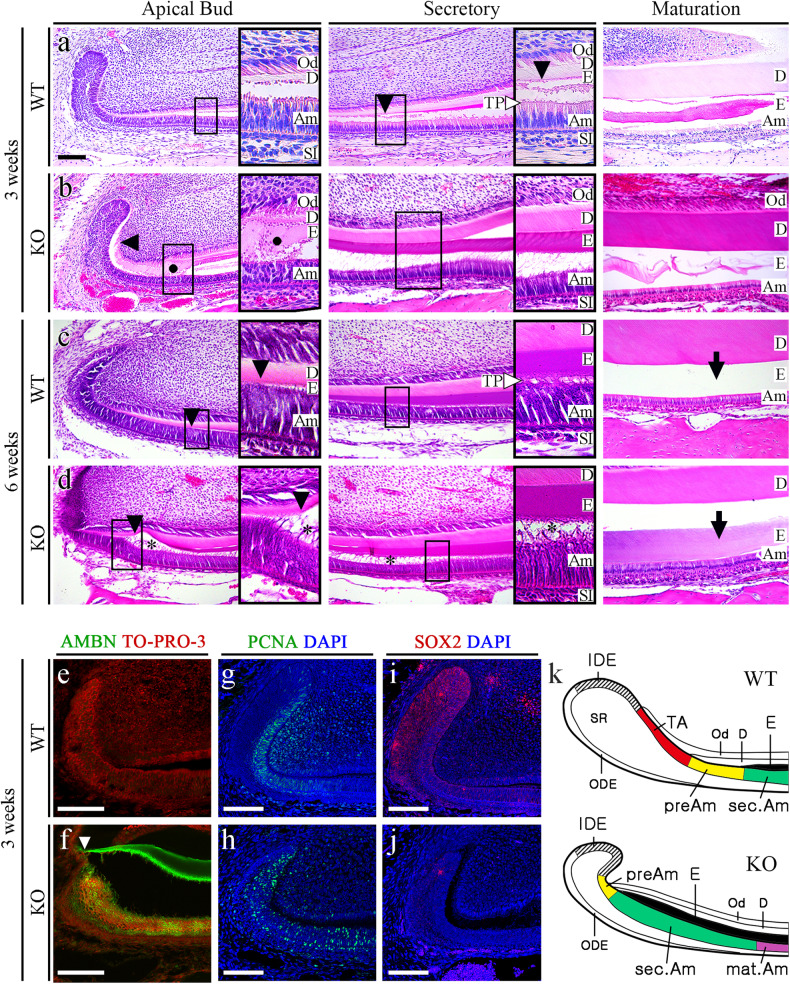
Reduced apical bud region size and accelerated ameloblast differentiation in *Mast4* KO incisors. **a**, **b** HE-stained images of incisors from 3-week-old WT and *Mast4* KO mice. The black arrowheads indicate the position of enamel deposition initiation. In terms of the amelogenesis stages, early enamel deposition was observed in the *Mast4* KO incisor compared to the WT incisor. **a** In the secretory and maturation stages, enamel and ameloblasts fully contacted each other via the Tomes’ process (white arrowhead). **b** In contrast to the WT incisor, the ectopic atypical enamel deposition in the *Mast4* KO incisor began at the apical region (black circle). In the maturation stage, hypomineralization was detected in the *Mast4* KO incisor. **c**, **d** HE-stained images of incisors from 6-week-old WT and *Mast4* KO mice. Early enamel deposition was also observed (black arrowheads). In the secretory stage, the Tomes’ processes of ameloblasts fully contacted the enamel in WT mice (white arrowhead), whereas the Tomes’ processes were stretched and destroyed through the apical bud region to the secretory stage region in KO mice (asterisks). After decalcification, **c** the enamel in the WT maturation stage contained a lower abundance of enamel matrix proteins than **d** the enamel in the *Mast4* KO maturation stage (arrows). (**d** maturation) The separation of enamel and dentin was an artifact that occurred during sample sectioning. **e**, **f** Ameloblastin (AMBN) localization in incisors from 3-week-old WT and *Mast4* KO mice. **f** AMBN was expressed and released to the enamel layer on the apical side in the *Mast4* KO incisor compared to the WT incisor. The white arrowhead indicates the position of enamel deposition initiation. **g**, **h** The localization of the TA cell marker PCNA in the apical bud region of WT and *Mast4* KO incisors. PCNA was localized densely in the TA region and adjacent mesenchyme in the WT but showed a scattered distribution in the *Mast4* KO apical bud. **i**, **j** The DESC marker SOX2 was expressed throughout the apical bud of the WT incisor and in a limited small area of the apical bud of the *Mast4* KO incisor. **k** Schematic diagrams of the mandibular labial incisor epithelium in WT and *Mast4* KO mice. In the mandibular incisors of *Mast4* KO mice, the size of the labial apical bud, including the SR and IDE, was reduced. The TA region disappeared, and the initiation of enamel deposition was shifted to the reduced apical bud in *Mast4* KO incisors. D dentin, E enamel, TP Tomes’ process, Am ameloblast, Od odontoblast, sec.Am secretory ameloblast, mat.Am maturative ameloblast, preAm preameloblast, IDE inner dental epithelium, ODE outer dental epithelium, SR stellate reticulum, TA transit-amplifying zone. All scale bars, 100 μm.

The apical buds of mouse incisors contain an epithelial stem cell niche that provides the inner dental epithelium (IDE) cells that differentiate into ameloblasts. To investigate stem cell differentiation, apical bud regions were dissected from the mandibles of 6-week-old WT and *Mast4* KO mice (Supplementary Fig. 8a, b). The initiation of enamel matrix secretion was shifted to the apical side of the *Mast4* KO incisors (Supplementary Fig. 8b). Interestingly, the apical bud of the *Mast4* KO incisor appeared to be reduced (Supplementary Fig. 8b, dotted line). In particular, the TA zone seemed to have disappeared. We investigated the histology of the incisor teeth in decalcified sagittal sections from WT and *Mast4* KO mice at 3 and 6 weeks of age (Fig. 2a–d). In WT mice, secretory stage ameloblasts were tall and columnar, and enamel matrix proteins were present in the forming enamel (Fig. 2a). During the maturation stage, WT mice exhibited characteristic shortened ameloblasts. In contrast, epithelial cells in the TA zone of the apical bud region appeared to have been transformed into secretory ameloblasts in the KO incisors (Fig. 2b). The initiation of enamel matrix secretion was shifted to the apical bud region in the *Mast4* KO incisors, while the initiation of secretion started in secretory stage ameloblasts in the WT incisors (Fig. 2a, b, arrowheads). In particular, ectopic atypical enamel matrix deposition without underlying dentin formation was detected in 40 to 50% of *Mast4* KO mice (Fig. 2b, circle). In the maturation stage, severe hypomineralization was detected in the *Mast4* KO incisor enamel (Fig. 2b Maturation). The initiation position of dentin matrix secretion did not differ between the WT and KO incisors. At 6 weeks, the initiation of enamel deposition in the KO incisors was shifted to the apical as observed at 3 weeks (Fig. 2c, d, arrowheads). Additionally, compared with the normal Tomes’ processes in WT mice (Fig. 2c, TP), the Tomes’ processes in *Mast4* KO mice were stretched through the apical bud region to the secretory region at 6 weeks (Fig. 2d, asterisks). The degradation and absorption of enamel matrix proteins, important functions of maturation stage ameloblasts, were performed properly, and the enamel space appeared empty in the WT incisors (Fig. 2c, arrow). However, the eosinophilic staining of the enamel space in *Mast4* KO incisors indicated that these functions were not performed properly in KO incisor ameloblasts and that significant amounts of enamel proteins remained (Fig. 2d, arrow). Enamel matrix proteins are substituted during enamel maturation, and the proportion of these proteins in mature enamel is known to be less than 2% m/m^42^. This feature of excessive remaining enamel proteins was consistent with the low mineral composition (high protein composition) and peeled enamel observed in *Mast4* KO mice (Fig. 1b, arrow and Supplementary Fig. 4). Ectopic enamel secretion was verified by evaluating ameloblastin expression (Fig. 2e, f), and ameloblastin was found to be expressed and released to the enamel layer on the apical side in *Mast4* KO incisors (Fig. 2f, arrowhead), suggesting that enamel matrix secretion is accelerated temporarily which resulted in advanced spatially in Mast4 KO incisors. Proliferating Cell Nuclear Antigen (PCNA), a TA cell marker in incisors, was expressed in the TA region of the epithelium and adjacent mesenchyme of the WT incisors, whereas PCNA was scattered throughout the epithelium and sparsely detected in the mesenchyme of the KO incisors (Fig. 2g, h). SOX2, which is a marker of dental epithelial stem cells (DESCs), was expressed throughout the WT apical bud, including in TA cells (Fig. 2i). However, SOX2 was detected in a limited area known as the stem cell niche in the apical bud of the KO incisors (Fig. 2i, j). Schematic diagrams showing these features of WT and *Mast4* KO incisors and a summary of the histological observations are presented (Fig. 2k).

**Fig. 3:**
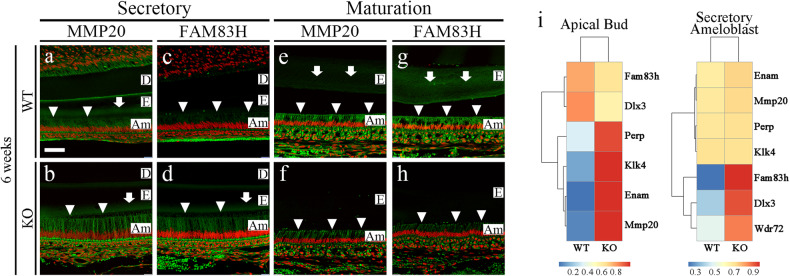
Enamel maturation dysregulation in *Mast4* KO incisors. **a**–**h** Enamel matrix protein (MMP20) and maturation-related protein (FAM83H) in the secretory and maturation regions of incisors from 6-week-old WT and *Mast4* KO mice. The arrowheads indicate ameloblasts expressing MMP20 and FAM83H. The arrows indicate the expression of enamel matrix proteins. **a**, **b** In the secretory region, the expression of MMP20 was similar between the WT and *Mast4* KO mice. **c**, **d** FAM83H expression was increased in *Mast4* KO ameloblasts. **e**–**h** In the maturation region, MMP20 and FAM83H expression was decreased in ameloblasts as well as in the enamel matrix in *Mast4* KO mice. **i** RNA-Seq analyses of apical buds and secretory ameloblasts from the incisors of 6-week-old WT and *Mast4* KO mice. At the apical bud, the expression of *Perp*, *Klk4*, *Enam* (*Enamelin*), and *Mmp20* was increased in *Mast4* KO incisors. In the ameloblast region, the expression of *Fam83h*, *Dlx3*, and *Wdr72* was increased in *Mast4* KO incisors. D dentin, E enamel, Am ameloblast. Scale bar; 50 μm.

To determine and identify additional molecules involved in the disrupted amelogenesis in *Mast4* KO mice, we screened MMP20 and FAM83H by immunofluorescence staining in 6-week incisors from WT and *Mast4* KO mice (Fig. 3a–h). Interestingly, the expression of MMP20, which aids in enamel protein alignment in the enamel matrix and ameloblasts, did not differ between WT and *Mast4* KO mice (Fig. 3a, b). However, FAM83H secretion into the enamel matrix was significantly increased in the *Mast4* KO secretory region (Fig. 3c, d). In the maturation region, the quantity of both proteins in the enamel matrix was lower in *Mast4* KO mice than in WT mice (Fig. 3e-h). In particular, the expression of FAM83H, which plays a key role in enamel maturation, shifted to an early stage in the secretory region (Fig. 3d, g, arrows and arrowheads). These results are consistent with the shifted initiation of enamel secretion and reduced size of the apical bud region in the *Mast4* KO incisors.

We further performed RNA sequencing analysis of two regions apical buds and secretory ameloblasts, to compare 6-week-old WT incisors and *Mast4* KO incisors (Fig. 3i). The major challenge can be considered to be the definition of the *Mast4* KO apical bud region (properties of secretory ameloblasts and apical buds) and the secretory ameloblast region (properties of maturative ameloblasts) due to the reduction in the apical bud size in *Mast4* KO mice. Consequently, transcriptome analysis of secretory ameloblasts (Fig. 3i) represented an area of an exact match, as shown in Fig. 3a–d. In the apical bud region, the expression of enamel matrix protein-encoding genes, including *Klk4*, *Enam*, and *Mmp20*, was increased in *Mast4* KO mice. In the secretory ameloblast region, the expression of enamel matrix maturation-related genes, including *Fam83h*, *Dlx3*, and *Wdr72*, was increased in *Mast4* KO mice. These results suggest that enamel secretion and maturation end at an early stage of amelogenesis in the *Mast4* KO incisor compared to the WT incisor.

### Effects of *Mast4* deficiency on the *Wnt* signaling pathway

**Fig. 4:**
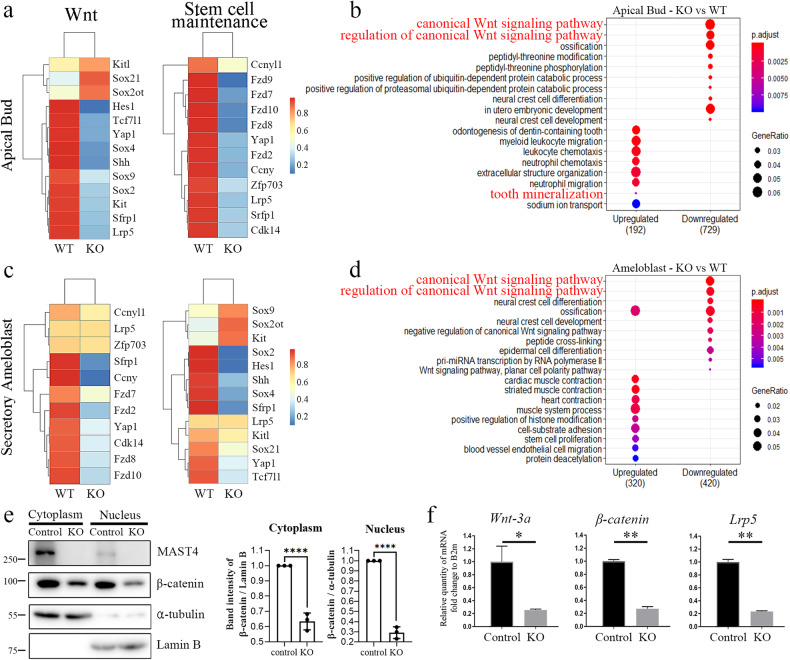
Disruption of stem cell maintenance and the *Wnt* signaling pathway in *Mast4* KO mice. **a**–**d** RNA-Seq analyses of apical buds and ameloblasts from both WT and *Mast4* KO incisors. **a**, **c** Heatmaps showing that canonical *Wnt*-related genes showed downregulation in *Mast4* KO apical buds and ameloblasts. The expression of genes related to stem cell maintenance was also downregulated in *Mast4* KO incisors. **b**, **d** GO analysis of both apical buds and ameloblasts revealed that the canonical *Wnt* signaling pathway was downregulated in *Mast4* KO incisors. **e** Subcellular fractionation of control mHat9d cells and mHat9d cells with lentivirus-mediated Mast4 KO was performed. Notably, β-catenin expression in both the cytoplasm and nucleus was decreased in *Mast4* KO cells (mean ± SD, *n* = 3). **f** Real-time PCR analysis of the expression of *Mast4*, *Wnt-3a*, *β-catenin*, and *Lrp5* in mHat9d cells (mean ± SD, *n* = 3). The expression of *Wnt*-related genes was reduced after *Mast4* KO in mHat9d cells. *p < 0.05, **p < 0.01, ****p < 0.0001.

Via RNA sequencing analysis of secretory ameloblasts and apical buds isolated from 6-week-old WT and *Mast4* KO incisors, changes in the *Wnt* signaling pathway profile and stem cell maintenance were detected. Changes in gene expression identified through analysis of differentially expressed genes (DEGs) were visualized on heatmaps, and large decreases in the expression of canonical *Wnt* signaling genes and stem cell maintenance-related genes were observed in both the apical bud and secretory ameloblast regions in *Mast4* KO incisors (Fig. 4a, c). Gene Ontology (GO) analysis was performed on the RNA sequencing data (Fig. 4b, d). Canonical *Wnt* signaling was significantly downregulated in both the apical buds and ameloblasts of *Mast4* KO mice. Interestingly, ossification was downregulated in *Mast4* KO ameloblasts. Some tooth mineralization markers were upregulated in apical buds (Fig. 4b). We confirmed these changes in the *Wnt* signaling pathway in *Mast4*-null mHat9d cells, a dental epithelial stem cell line derived from the apical bud epithelium of a mouse incisor, finding that β-catenin expression was decreased in both the nucleus and cytoplasm (Fig. 4e). *Mast4* ablation markedly decreased the transcription of *Wnt* signaling molecules, including *Wnt-3a*, *β-catenin*, and *Lrp5* (Fig. 4f). This result suggests that ameloblast differentiation is accelerated due to altered *Wnt* signaling and stem cell maintenance in the apical bud region.

### MAST4 promoted the nuclear translocation of DLX3 by mediating serine/threonine phosphorylation of DLX3 within the nuclear localization signal (NLS)

**Fig. 5:**
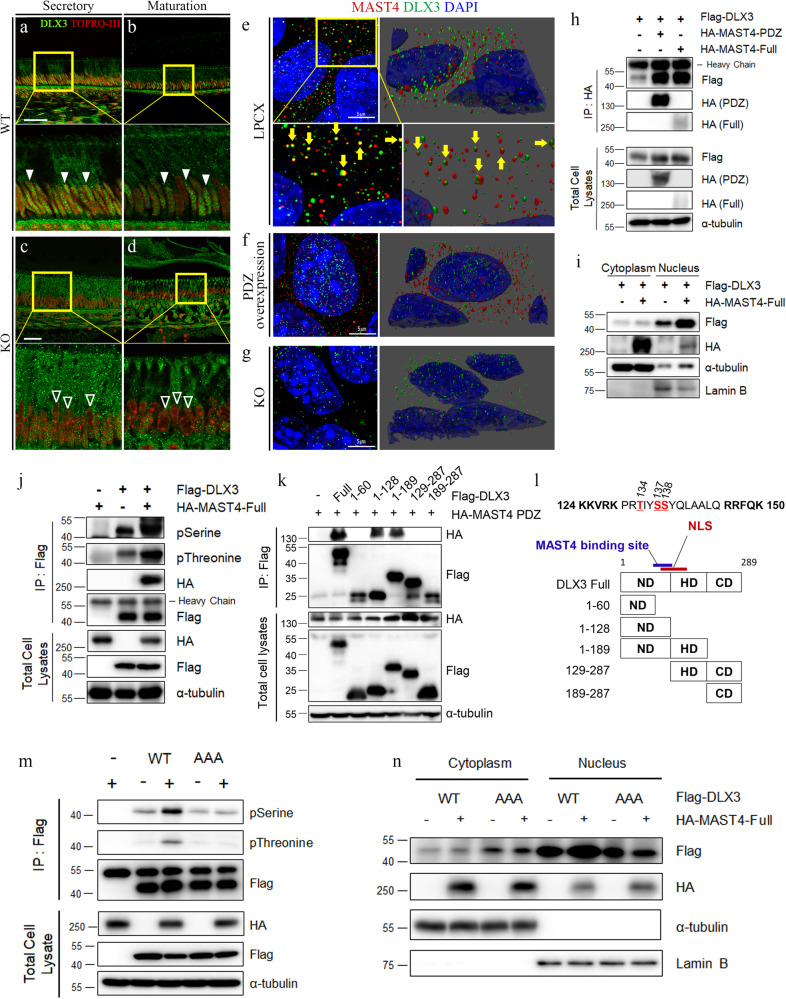
MAST4 regulates the translocation of DLX3 by mediating its phosphorylation near the NLS region. **a**–**d** DLX3 localization in incisors from 6-week-old WT and *Mast4* KO mice. **a**, **b** In the WT incisor, DLX3 localized to the nucleus (arrowheads) and cytoplasm. **c**, **d** In the *Mast4* KO incisor, the abundance of DLX3 in the nucleus was lower than that in the WT incisor (blank arrows). **e**–**g** MAST4 and DLX3 localization in empty LPCX vector-transfected control (**e**), MAST4 PDZ-overexpressing (**f**), and MAST4 KO (**g**) mHAT9d cells. Immunocytochemistry was performed, and confocal z-stack images were acquired in ultra-high-resolution mode (Lightning mode in Leica operating software). 3D reconstructions of the images were generated. The yellow arrows indicate direct binding of MAST4 and DLX3 in (**e**). **h** Immunoprecipitation was conducted after transient transfection of both HA-MAST4 PDZ and HA-MAST4-Full into HEK293T cells. **i** Analysis of DLX3 expression using subcellular fractionation following transient transfection of full-length MAST4 in the HEK293T cell line. The expression of α-tubulin in the cytoplasm and Lamin B in the nucleus served as controls for the efficiency of subcellular fractionation. **j** Flag-DLX3 was immunoprecipitated, and the complexes were analyzed by western blotting. Note that DLX3 phosphorylation was increased in the presence of HA-MAST4-Full in the HEK293T cell line. **k** HA-MAST4 PDZ and various *Dlx3* deletion mutants were cotransfected into HEK293T cells, followed by immunoprecipitation. Note that MAST4 bound to the C-terminus of the ND. **l** Schematic diagram showing the deletion of the entire *Dlx3* sequence. Note that the NLS is located at aa 124–150. **m** HEK293T cells were transiently cotransfected with Flag-DLX3^WT^, the DLX3^AAA^ mutant and HA-MAST4-Full. Flag-DLX3 was immunoprecipitated, and the complexes were analyzed by western blotting. Note that DLX3 phosphorylation was increased in the presence of MAST4. **n** Flag-DLX3^WT^, the DLX3^AAA^ mutant and HA-MAST4-Full were transiently cotransfected into HEK293T cells, and subcellular fractionation was performed. Notably, compared with that of DLX3^WT^, the proportion of the DLX3^AAA^ mutant in the cytoplasm was higher and was not regulated by MAST4 (mean ± SD, *n* = 3). NLS nuclear localization site, ND N-terminal domain, HD homeodomain, CD C-terminal domain. Scale bars; **a**–**d**, 40 μm; e–g, 5 μm. The data were representative of three independent experiments.

Considering that the phenotypes of enamel disruption and the significant reduction in the mineral content found in *Mast4* KO mice were similar to the phenotypes observed in conditional *Dlx3* KO mice^30^, we focused on examining the relationship between MAST4 and the DLX3 transcription factor, which plays an essential role in amelogenesis. First, we checked the distribution pattern of DLX3 in WT and *Mast4* KO mice. The expression of DLX3 showed distinct localization patterns depending on the differentiation stage in the incisors of 6-week-old WT mice. DLX3 was predominantly located in the nuclei of ameloblasts at the secretory stage in the incisors of WT mice (Fig. 5a, arrowheads). At the maturation stage, DLX3 expression remained high in the nuclei of ameloblasts (Fig. 5b, arrowheads). However, in the incisors of *Mast4* KO mice, DLX3 localization to the nucleus was decreased at both the secretory and maturation stages (Fig. 5c, d, blank arrowheads). These results suggest that the nuclear translocation of DLX3 might be impaired in *Mast4* KO mice.

Based on this observation, we examined whether MAST4 regulates the nuclear translocation of DLX3. Because of the high molecular weight (>285 kDa) of full-length MAST4 and its relatively low expression in mHat9d cells, we used a truncated *Mast4* construct (MAST4 PDZ) containing the DUF, kinase, and PDZ domains that was shown to function normally in two previous studies^37,38^. To explore the relationship between MAST4 and DLX3, immunocytochemistry was performed for MAST4 and DLX3 in control, MAST4 PDZ-overexpressing, and MAST4-depleted mHAT9d cell lines. In the control (empty LPCX vector-transfected) cells, several MAST4 and DLX3 proteins directly bound to each other in the cytoplasm (Fig. 5e, yellow arrows and Supplementary Fig. 9a, d). Some DLX3 was detected in the nucleus, while most MAST4 was located in the cytoplasm. In MAST4 PDZ-overexpressing mHAT9d cells, DLX3 was translocated into the nucleus, and the abundance of MAST4 was increased in the cytoplasm (Fig. 5f and Supplementary Fig. 9b, e). A direct interaction between MAST4 and DLX3 was observed in these cells, but the number of complexes was decreased compared to that in the control cells. In MAST4-depleted cells, the abundances of MAST4 and DLX3 in the cytoplasm were not decreased (Fig. 5g and Supplementary Fig. 9c, f). The cross-sectional z-stack images acquired from these three cell types (LPCX, PDZ over, and KO), along with the related quantitative analysis, demonstrated a positive correlation between the MAST4 level and the degree of DLX3 nuclear translocation (Supplementary Fig. 9a–c, g). Additionally, an immunoprecipitation assay was performed in HEK293T cells, and interaction between MAST4 (both MAST4 PDZ and MAST4-Full) and DLX3 was detected (Fig. 5h). We next confirmed that DLX3 nuclear translocation increased when MAST4 was stably overexpressed in mHat9d cells (Supplementary Fig. 10a) or transiently overexpressed in HEK293T cells (Fig. 5i and Supplementary Fig. 10b). Considering that MAST4 functions as a serine/threonine kinase and that DLX3 is targeted by MAST4 for phosphorylation to regulate its DNA binding activity^32^, we examined whether MAST4 induces DLX3 phosphorylation. Interestingly, immunoprecipitation assays revealed that MAST4 overexpression significantly increased both the serine and threonine phosphorylation of DLX3 in both mHat9d (Supplementary Fig. 10e) and HEK293T cells (Fig. 5j and Supplementary Fig. 10c, d, f).

Next, to determine the sites of MAST4-mediated phosphorylation, multiple *Dlx3* deletion mutants were generated, and we found that MAST4 bound to the C-terminus of the ND of DLX3, which is adjacent to the NLS region (aa 124–150) (Fig. 5k, l). In a previous report, it was revealed that the NLS region of DLX3 is a critical region for its nuclear translocation^43^. In addition, the mechanism by which phosphorylation adjacent to the NLS region regulates protein translocation has been elucidated by performing serine-to-alanine mutations of the target residues^44,45^. Therefore, to investigate whether MAST4-mediated phosphorylation adjacent to the NLS of DLX3 is necessary for its nuclear translocation, we generated a nonphosphorylatable mutant with alanine substitutions of the serine and threonine residues (T134, S137, and S138) within the NLS region (DLX3^AAA^, Supplementary Fig. 16a). Interestingly, while MAST4 overexpression significantly increased serine and threonine phosphorylation of DLX3^WT^, the phosphorylation of the DLX3^AAA^ mutant was not affected by MAST4 overexpression in HEK293T cells (Fig. 5m and Supplementary Fig. 11). More DLX3^AAA^ than DLX3^WT^ was localized to the cytoplasm, but the nuclear translocation of DLX3^AAA^ was not promoted by MAST4 (Fig. 5n and Supplementary Fig. 12). Furthermore, we confirmed that the expression of carbonic anhydrase genes, such as *Car6* and *Car12*, and ion transporter genes, such as *Slc26a1* and *Slc34a2*, in *Mast4* KO mHat9d cells was rescued through the overexpression of DLX3 (Supplementary Fig. 13). These results suggest not only that phosphorylation of the DLX3 NLS by MAST4 is important for the nuclear translocation of DLX3 but also that the abnormal enamel secretion and maturation phenotypes observed in *Mast4* KO mice are mediated through the regulation of DLX3 by MAST4.

Considering the previously reported correlation between DLX3 and *Wnt* signaling, we investigated whether the alterations in *Wnt* signaling observed in *Mast4* KO mice are associated with the abnormal translocation of DLX3. Interestingly, in mHat9d cells, transient overexpression of DLX3 did not differentially regulate *Wnt* signaling, and DLX3 did not exhibit any specific effect on the inhibition of *Wnt* signaling caused by Mast4 KO (Supplementary Fig. 14). Therefore, further research is needed to elucidate the relationship between DLX3 and *Wnt* signaling. Additionally, we confirmed that the changes in *Wnt* signaling and DLX3 activity observed in Mast4 KO mice were independent of each other.

### Phosphorylation of the DLX3 NLS by MAST4 regulated the activation of DLX3 target genes involved in pH regulation

**Fig. 6:**
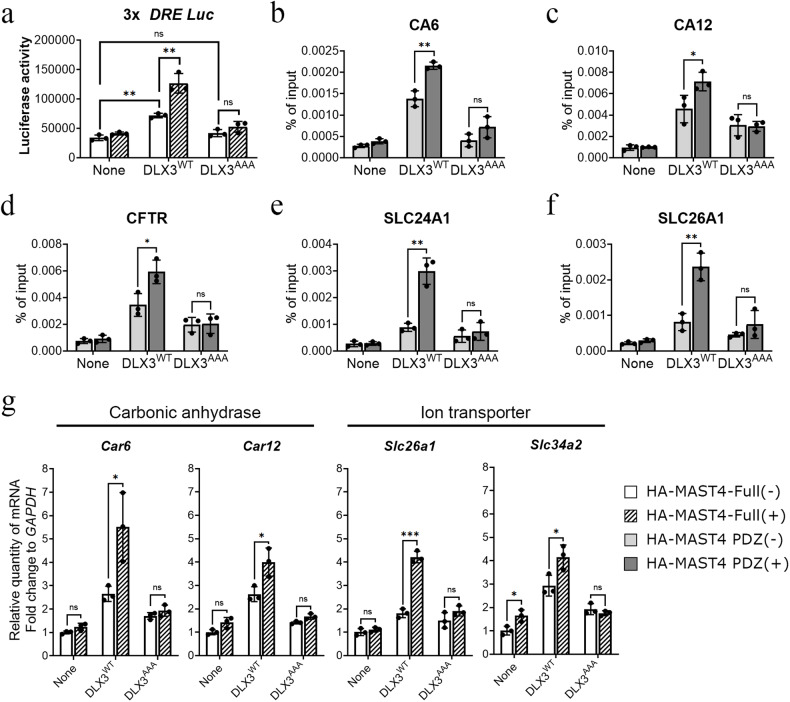
The phosphorylation of the DLX3 NLS by MAST4 regulates the activation of target genes involved in pH regulation. **a** 3xDRE-luc, DLX3, and HA-MAST4-Full were transiently overexpressed in HEK293T cells, and beta-galactosidase was cotransfected for normalization. Luciferase activities were measured after 48 h. **b**–**f** DLX3^WT^, DLX3^AAA^, and HA-MAST4 PDZ were transiently transfected into HEK293T cells. ChIP assays showed that transfection of DLX3^WT^ increased target gene promoter binding and that HA-MAST4 PDZ cotransfection further increased target gene promoter binding, whereas transfection of DLX3^AAA^ had no significant effect. **b**–**d** Carbonic anhydrases. **e**, **f** Ion transporters. **g** RT‒qPCR results for carbonic anhydrases and ion transporters involved in pH regulation. mHat9d cells were transiently transfected with DLX3^WT^, DLX3^AAA^, and HA-MAST4-Full. The data were presented as the means ± SDs (*n* = 3 for **a**–**g**). *p < 0.05; **p < 0.01; ns nonsignificant.

To understand the functional implications of phosphorylation of the NLS of the DLX3 transcription factor, a luciferase reporter assay using pGL3-3xDRE, which contains three copies of the DLX3-responsive elements, was performed to assess the transcriptional activity of both DLX3^WT^ and DLX3 mutants in HEK293T cells^46^. As expected, the basal transcriptional activity of DLX3^WT^ was greater than that of the DLX3^AAA^ mutant (Fig. 6a and Supplementary Fig. 15). In particular, the transcriptional activity of DLX3^WT^ was further increased when MAST4 was overexpressed, whereas the effect of MAST4 overexpression on the transcriptional activity of the DLX3^AAA^ mutant was relatively not significant. In addition, the phosphomimetic DLX3^EEE^ mutant with glutamic acid substitutions further increased the basal transcriptional activity of DLX3 without being affected by MAST4 overexpression, indicating that the phosphorylation status of the NLS of DLX3 is critical for activation of its target genes (Supplementary Fig. 16b). Next, we investigated whether the occupancy of each target gene promoter by DLX3 is regulated by MAST4-mediated NLS phosphorylation. In a previous report, direct target genes of DLX3, such as carbonic anhydrase and ion transporter genes involved in pH regulation, were identified, and DLX3 binding sites in the promoter of each target gene were also identified^30^. With reference to a previous report, chromatin immunoprecipitation (ChIP) assays were conducted to examine the binding of DLX3 to its target gene promoters in both HEK293T and mHAT9d cells. Interestingly, in the case of carbonic anhydrase genes (*CA6* and *CA12****)*** and ion transporter genes (*CFTR, SLC24A1*, and *SLC26A1*), DLX3^WT^ exhibited increased occupancy compared with that of DLX3^AAA^ (Fig. 6b–f and Supplementary Fig. 17). In particular, the occupancy of DLX3^WT^ on the target gene promoters was further increased by MAST4 PDZ overexpression, while that of DLX3^AAA^ was not affected. Next, to confirm whether the mRNA levels of the target genes are regulated by altered translocation of DLX3 via MAST4, RT‒qPCR was performed in mHat9d cells. The activation of the carbonic anhydrase target genes *Car6* and *Car12* and the ion transporter target genes *Slc26a1* and *Slc34a2* was significantly downregulated by the expression of DLX3^AAA^, while expression of the DLX3^EEE^ mutant further increased target gene activation (Fig. 6g and Supplementary Fig. 16c). Consistently, a MAST4 PDZ-mediated increase in target gene activation was observed when DLX3^WT^ was transiently co-overexpressed, while neither of the two DLX3 mutants was further differentially regulated, confirming the NLS phosphorylation site-specific role of Mast4. These results indicate that phosphorylation of the DLX3 NLS by MAST4 plays an important role in promoting the nuclear translocation of DLX3 and subsequent activation of the target genes.

## Discussion

*Mast4* KO mice showed abnormal enamel formation in incisor teeth. Specifically, enamel opacity was increased in the mandibular incisors of *Mast4* KO mice, while there was reduced enamel hardness in *Mast4* KO mice compared to WT mice. During dentinogenesis, odontoblasts secrete protein components of an unmineralized, collagen-rich extracellular matrix termed predentin. Later, the predentin is transformed into a mineralized tissue when apatite crystals are deposited within and around collagen fibrils^47^. After dentin matures, enamel can be deposited on the dentin surface by ameloblasts^48^. MAST4 seems to regulate amelogenesis at this time point. Excessive and ectopic enamel matrix protein secretion was found at sites without underlying dentin formation in *Mast4* KO incisors. Additionally, enamel maturation was retarded, causing mechanical weakness of the incisors. However, there were no differences in aspects related to dentinogenesis, including odontoblast differentiation, the initiation location of dentin secretion, and dentin thickness.

Based on SEM analysis and EPMA, the grid shape of the enamel rod array was collapsed, and the mineral content was insufficient. Hardness testing also showed that the overall enamel quality was inferior, and this inferiority may be caused by a failure in the mechanism maintaining the stemness of stem cells, which are known to exist in the apical bud. This speculation was confirmed by investigating the expression of the transcription factor SOX2, which is known to be essential for stem cells and progenitor cells to maintain pluripotency^49^ and for DESC maintenance and proliferation^18^. This finding was supported by the observation that this abnormality was not found at the embryonic or neonatal stage, appearing only at postnatal week 2, and no disruption was found in relation to the molar dentition, which does not require stem cell maintenance. Failure of appropriate stem cell maintenance may alter or accelerate the differentiation of inner dental epithelial cells to the secretory ameloblast stage in the apical bud region. Enamel matrix secretion was also accelerated in turn, and the discrepancy of maturation timing was thought to cause physicochemical problems.

The present study suggests that MAST4 is a key factor associated with stem cell maintenance by regulating the *Wnt* signaling pathway and maturation through the control of DLX3 activity. Our previous study revealed that Mast4 regulates *Wnt* signaling by phosphorylating β-catenin^37^. Another of our previous studies revealed failure of spermatogonial stem cell maintenance in *Mast4* KO testes^50^. In addition, it has been reported that DLX3 is a downstream target of the *Wnt* signaling pathway in hair follicle development^24^. Several previous studies have shown that DLX3 regulates enamel matrix protein secretion by functioning as a matrix protein transcription factor^29,30^ and by regulating DKK1, which is a *Wnt* signaling inactivator^22,51^. Interestingly, our RNA sequencing data revealed that *Mast4* regulated not only *Dlx3*-related genes but also canonical *Wnt* signaling-related genes. Numerous studies have shown that *Wnt*/β-catenin signaling is closely related to stem cell self-renewal and maintenance in several organs^52–55^. In addition, considering that no abnormalities were found in either neonatal incisor ameloblasts or in molars where adult stem cells do not exist after their development is completed at the fetal stage, it can be inferred that MAST4 is involved in the maintenance of adult stem cells via several signaling pathways.

At the amelogenesis stage, it is well known that DLX3 also regulates the transcription of enamel matrix-related genes. However, it was reported that the transcription of DLX3 target genes related to the enamel matrix was not significantly altered in a study using *Dlx3* conditional KO mice, although these mice still exhibited the AI phenotype^30^. In our study, the mRNA expression levels of enamel matrix proteins did not change significantly, suggesting that other DLX3 targets, such as genes involved in pH regulation during ameloblast maturation, are critical for the AI phenotype. Our results demonstrate that MAST4 interacts with DLX3 and regulates its transcriptional activity, which may ultimately affect incisor amelogenesis. In addition, the nuclear localization of various proteins with an NLS is regulated by phosphorylation of the NLS^56^. We confirmed that MAST4 directly binds to DLX3 and phosphorylates three residues located in the NLS, ultimately increasing both nuclear translocation and target gene activation. The finding that the genes involved in pH regulation are specifically regulated by MAST4 and DLX3 is highly correlated with the abnormal ion distribution shown in previous EPMA results in *Mast4* KO mice (Supplementary Fig. 5). Additional evidence gained using a series of NLS phosphorylation site mutants of Dlx3 strengthened the idea of the importance of the NLS phosphorylation status in the regulation of DLX3 target gene activation and DLX3 nuclear translocation. Overall, the mislocalization of DLX3 due to the loss of MAST4 prevented ameloblast maturation, which requires appropriate pH regulation, ultimately resulting in the AI phenotype. These previous results and our results suggest that MAST4 is a putative candidate involved in controlling the activity of DLX3.

**Fig. 7:**
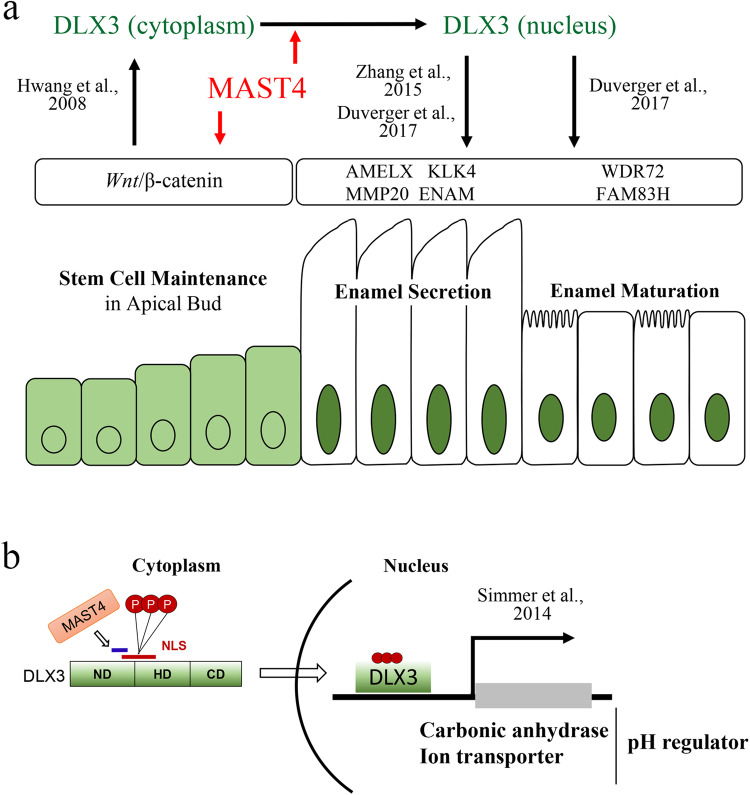
MAST4 spatiotemporally regulates amelogenesis in different ways. **a** MAST4 functions as a stem cell maintenance mediator in the incisor apical bud. Hwang et al. reported that DLX3 is a downstream target of the *Wnt* signaling pathway. Our RNA sequencing data showed that canonical *Wnt* signaling-related genes were downregulated in *Mast4* KO incisor tissues. MAST4 functions as a kinase of DLX3 in ameloblasts during enamel maturation by controlling DLX3 nuclear localization. *Dlx3* is an amelogenesis imperfecta (AI)-related gene. Zhang et al. and Duverger et al. reported that DLX3 regulates the secretion of enamel matrix proteins and enamel maturation proteins by functioning as a transcription factor for those matrix proteins. RNA sequencing data showed that *Mast4* regulated *Dlx3*-related genes related to the enamel matrix and maturation. **b** DLX3 controls the expression of pH regulators, one of the critical factors of enamel maturation. Simmer et al. reported that DLX3 regulates the expression of carbonic anhydrases and ion transporters, which are essential for enamel maturation. Luciferase assay and ChIP analysis data showed that carbonic anhydrases and ion transporters were downregulated in *Mast4* KO incisor tissues. The spatiotemporal regulation of *Wnt* and DLX3 localization by MAST4 is a key mechanism of stem cell maintenance, differentiation, and acquisition of physical properties of ameloblast products.

Considering that DLX3 is a transcription factor, its distribution appears to affect enamel matrix protein secretion via translocation to the nucleus during the secretory stage. The finding that MAST4 is involved in the nuclear localization of DLX3 also supports this hypothesis. A summary of the relationships between MAST4 and DLX3 is shown in Fig. 7. Despite the nuclear localization of DLX3 in mHat9d cells, the cause of decreased FAM83H and ameloblastin expression was not revealed in this study. Since mHat9d cells, a cell line derived from the apical bud were used, our findings suggest that DLX3 may exert different effects depending on the degree of differentiation. Specifically, transcriptome analysis in a previous study revealed that the expression of genes involved in pH regulation (*Cftr*, *Slc24a4*, *Slc26a7*, *Slc34a2*, and *Slc39a2*) increased significantly from the secretory stage to the maturation stage and that the expression of *Mast4* also increased 3.2-fold^57^, suggesting the need for a more detailed study of Mast4 expression patterns during ameloblast maturation. In addition, DLX3 is known to be related to dentinogenesis^58,59^. However, *Mast4* ablation did not result in abnormal odontoblast differentiation or dentin formation, suggesting that further studies on mesenchymal cell differentiation, including dentinogenesis and ectopic enamel matrix deposition in the absence of underlying dentin, are needed.

In conclusion, MAST4 plays a key role in the maintenance of stem cells and the regulation of differentiation by regulating the *Wnt* signaling pathway. Ablation of Mast4 also causes accelerated amelogenesis in the incisor tooth, improper enamel maturation, and abnormal physical properties. These phenomena are triggered by the regulation of DLX3 nuclear localization by MAST4. These findings suggest a novel mechanism for controlling the transcriptional activity of DLX3. MAST4 is closely associated with the entire amelogenesis process in the mouse incisor.

### Supplementary information

Supplementary inforamation

## Data Availability

The raw RNA-Seq datasets were generated in FASTQ format. A total of four sets of raw data can be downloaded from the Sequence Read Archive (SRA) under BioProject accession number PRJNA785577.
