# Supplementary material for: MAST4 regulates stem cell maintenance with DLX3 for epithelial development and amelogenesis
Source: Exp Mol Med. 2024 Jul 1;56(7):1606–19. doi: 10.1038/s12276-024-01264-5 (PMC11297042; doi:10.1038/s12276-024-01264-5)
Supplement: Supplementary file 1 — Supplementary inforamation [file 12276_2024_1264_MOESM1_ESM.pdf]

MAST4 regulates stem cell maintenance with DLX3 for epithelial development and amelogenesis

Dong-Joon Lee, Pyunggang Kim, Hyun-Yi Kim, Jinah Park, Seung-Jun Lee, Haein An, Jin Sun Heo, Min-Jung Lee, Hayato Ohshima, Seiya Mizuno, Satoru Takahashi, Han-Sung Jung, and Seong-Jin Kim

Han-Sung Jung (Lead contact)

Address: #601, Department of Oral Biology, Yonsei University College of Dentistry, 50-1, Yonsei-ro, Seodaemun-gu, Seoul, 03722, Korea

Email: hsj8076@gmail.com

Seong-Jin Kim

Address: GILO Institute, 92, Myeongdal-ro, Seocho-gu, Seoul, 06668, Korea

Email: jasonsjkim@gilo.or.kr

Extended Methods

Subcellular fractionation and Western blot

Virus-infected cells were lysed in RIPA buffer containing a protease inhibitor cocktail (cOmplete; #11697498001, Roche, IN, USA). Nuclear and cytoplasmic fractions of mHat9d cells were performed using the NE-PER Nuclear and Cytoplasmic Extraction reagents (Thermo Scientific), according to the manufacturer's protocols. Cell extracts were fractionated by SDS-PAGE transferred to a polyvinylidene difluoride membrane using a transfer apparatus according to the manufacturer's protocols (Bio-Rad). After incubation with 3% BSA in TBST (10 mM Tris, pH 7.4, 150 mM NaCl, 0.1% Tween 20) for 60 min, the membrane was incubated with antibodies against anti-β-catenin (SC-7199, Santa Cruz Biotechnology, Inc., USA; dilution 1:2000), anti-Histone H3 (ab4729, Abcam, UK; dilution 1:200) and anti-GAPDH (SC-32233, Santa Cruz Biotechnology, Inc., USA; dilution 1:100), anti-Flag (F3165; Sigma-Aldrich, USA; 1:5000), anti-HA (sc-7392, Santa Cruz Biotechnology, USA; 1:1000), anti-Lamin B (SC-374015, Santa Cruz Biotechnology, Inc., USA; 1:1000) and anti-α-tubulin (T5168, Sigma-Aldrich; 1:3000) at 4 °C overnight. Membranes were washed six times for 10 min and incubated with HRP-conjugated secondary antibodies for 2h. Blots were washed six times with TBST and developed with the ECL system (RPN2232, GE Healthcare Life Sciences, USA) according to the manufacturer's protocols.

Immunohistochemistry

Samples were fixed in 4% paraformaldehyde in phosphate buffered saline (PBS), decalcified in 10% EDTA (pH 7.4; BE021, Bio-solution co. Ltd., Korea) for 48 h at 50°C and then embedded in paraffin using standard procedures. Sections (6-μm thickness) of the specimens were incubated in 10 mM citrate buffer (pH 6.0) overnight at 60°C or Proteinase K (10 μg/ml, AM2546, Thermo Fisher Scientific, MA, USA) for 20 min at 37 ℃. The specimens were incubated with anti-MAST4 (BS5791, Bioworld Technology, Inc., MN, USA; dilution 1:150), anti-AMBN (orb155652, Biorbyt, UK; dilution 1;200), anti-PCNA (ab152112, Abcam, UK; dilution 1:200), anti-SOX2 (ab137385, Abcam, UK; dilution 1:200), anti-DLX3 (PA5-40506, Invitrogen, OR, USA; dilution 1:100), anti-MMP20 (ab198815, Abcam, UK; dilution 1:100), anti-FAM83H (NBP1-93737, Novus Biologicals, USA; dilution 1:50) antibodies at 4°C overnight. The specimens were incubated with goat anti-rabbit Alexa Fluor 488 (A11008, Invitrogen, OR, USA; dilution 1:200) or goat anti-mouse Alexa Fluor 488 (A11001, Invitrogen, OR, USA; dilution 1:200). Then sections were stained with TO-PRO™-3 (T3605, Invitrogen, OR, USA; dilution 1:1000), or DAPI (D1306, Invitrogen, OR, USA; 30nM) for nucleus and examined using a confocal laser microscope (Dmi8, Leica, Germany).

Immunoprecipitation (IP)

Cells were lysed with RIPA buffer containing protease inhibitor cocktail (Complete; Roche). For immunoprecipitation, protein extracts were incubated overnight with the indicated primary antibodies at 4℃. Dynabeads Protein G (Invitrogen) was used to precipitates antibody-bound proteins. Samples were separated by SDS-PAGE and electro-transferred to a polyvinylidene difluoride membranes (PVDF; Millipore). The membrane was blocked at room temperature for 1h and incubated with the indicated primary antibodies overnight at 4°C. The primary antibodies used were as follows: phospho-Serine (P5747, Sigma-Aldrich; 1:1000), phospho-Threonine (#9386, Cell signaling; 1:1000), DLX3 (PA5-40506, Invitrogen, OR, USA), HA (F-7, Santa

Cruz Biotechnology), Flag (F3165; Sigma-Aldrich, USA; 1:5000) and α-tubulin (T5168, Sigma-Aldrich; 1:3000). Horseradish peroxidase-conjugated antibodies (Millipore) were used as secondary antibodies. The peroxidase reaction products were visualized with WESTZOL (Intron). All signals were detected by Amersham Imager 600 (GE Healthcare Life Sciences).

Real-Time quantitative PCR (RT-qPCR)

For the RT-qPCR, the total RNA of the cells was extracted using TRIzol reagent (Invitrogen, Carlsbad, CA). The extracts were reverse transcribed using Maxime RT PreMix (#25081, iNtRON, Korea). RT-qPCR was performed using a StepOnePlus Real-Time PCR System (Applied BioSystems, USA). The amplification program consisted of 40 cycles of denaturation at 95°C for 15 s and annealing at 60 ℃ for 30 s. The expression levels of each gene are expressed as normalized ratios against the *B2m* housekeeping gene. The oligonucleotide primers for RT-qPCR are described in Supplementary Table 1.

Chromatin IP (ChIP) assay

Cells were cross-linked with 4% paraformaldehyde for 10 minutes at room temperature. Glycine was added to a final concentration of 125 mM for 5 minutes to quench the formaldehyde crosslinks. Cells were washed with ice-cold phosphate buffered saline, harvested by scraping, pelleted, and resuspended in SDS lysis buffer (50 mM Tris-HCl [pH 8.1], 1% SDS, 10 mM EDTA) with complete protease inhibitor cocktail (Roche). Cell extracts were sonicated with a Bioruptor TOS-UCW-310-EX (output, 250W; 25 cycles of sonication with 30-seccond intervals; Cosmo Bio). Samples were centrifuged at 14,000 rpm at 4℃ for 10 minutes, and the supernatants were diluted 10-fold in dilution buffer (20 mM Tris-HCl [pH 8.0], 2 mM EDTA, 1% Triton X-100, 150 mM NaCl, and complete protease inhibitor cocktail). Chromatin samples were precleared with protein A-agarose beads (Santa Cruz) for 2h before immunoprecipitation against Flag (Sigma-Aldrich) antibodies overnight at 4 ℃. Immune complexes were collected with protein A-agarose beads. Samples were washed five times (first wash with low salt immune complex wash buffer [20 mM Tris-HCl, pH.8.0, 2 mM EDTA, 1% Triton X-100, 0.1 % SDS, and 150 mM NaCl], second wash with high salt immune complex wash buffer [20 mM Tris-HCl, pH.8.0, 2 mM EDTA, 1% Triton X-100, 0.1% SDS, and 500 mM NaCl], third wash with LiCl immune complex wash buffer [10 mM Tris-HCl, pH.8.0, 1 mM EDTA, 250 mM LiCl, 1% NP-40, and 1% Na-deoxycholate], and the last two washes with TE buffer). Immunoprecipitated samples were eluted with buffer containing 1% SDS and 100 mM NaHCO3 at room temperature. Eluates were heated overnight at 65℃ to reverse crosslinks after adding NaCl to a final concentration of 100 mM. Genomic DNA was extracted with a PCR purification kit (GeneAll). Precipitated chromatin by real-time PCR and the readouts were normalized using 5% input chromatin for each sample. The experiments were repeated two or more times.

Primers for ChIP-PCR.

**Gene**	Forward (5’-3’)	Reverse (5’-3’)
CA6	AGC AAA GGG TGG TTT AGC C	AGG AAC AGG GAC AGC AGA AG
CA12	GCT ACA ACT CAC CTA GGA TCT GG	CTT CGC TCT CCT GGC TTC

CFTR	GTG CGT AGT GGG TGG AGA AA	CCC TTC CTT TTG CTC TTT CC
SLC24A1	GAG GGA ACA ACG CAC ATT CT	CCC AGT CTC TGC TTT CAA GG
SLC26A1	TCC TAC AAG CCC CTG ATT ACA	GTC TGT GCC CTG GAC TCT G
AMELX	TTT CTT TCT CTG CAT TTC TTT T	GAC AAG CAC TTT GTT GCA TT
ENAM	TAG GTT TCA GCT CCC AGG TT	TGA GTT TTT CCC CCT TTA CTT

Site-directed mutagenesis

Using the Flag-tagged wild-type *Dlx3* plasmid (Flag-DLX3 or DLX3^{WT}) as a template, PCR-based mutagenesis was performed using a primer containing the desired mutation site (T134, S136, and S137). We generated mutations that mimicked either inactive phosphorylation status (serine/threonine to alanine) or activated phosphorylation status (serine/threonine to glutamic acid). The completed PCR product was cut with DpnI for 2 h and transformed into E. coli DH5alpha competent cells by heat shock. Colonies from each plate were grown and DNA was extracted. Mutations were verified by sequencing.

Luciferase assay

The HEK293T cells were transiently transfected with 3X DRE-Luc and HA-MAST4-Full, 3Flag-DLX3 and series of mutation constructs using polyethylenimine (Polysciences, Inc). After 24 h, cells were lysed and the luciferase activities were analyzed using the Luciferase Assay System kit (Promega) according to the manufacturer’s protocol. All assays were done in triplicate, and all values were normalized for transfection efficiency against β-galactosidase activities.

Supplementary Figures

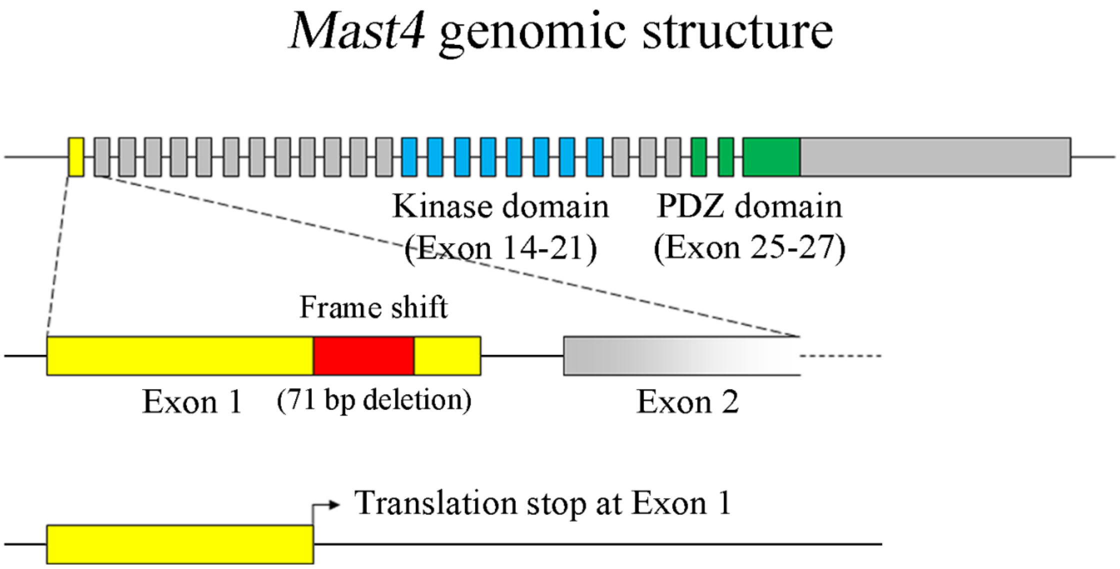

Supplementary Fig. 1. Structure of the *Mast4* KO allele.

The mouse *Mast4* locus is depicted with exons as boxes. A new stop codon is generated in the first exon of mRNA after the 71 bp nucleotides are deleted.

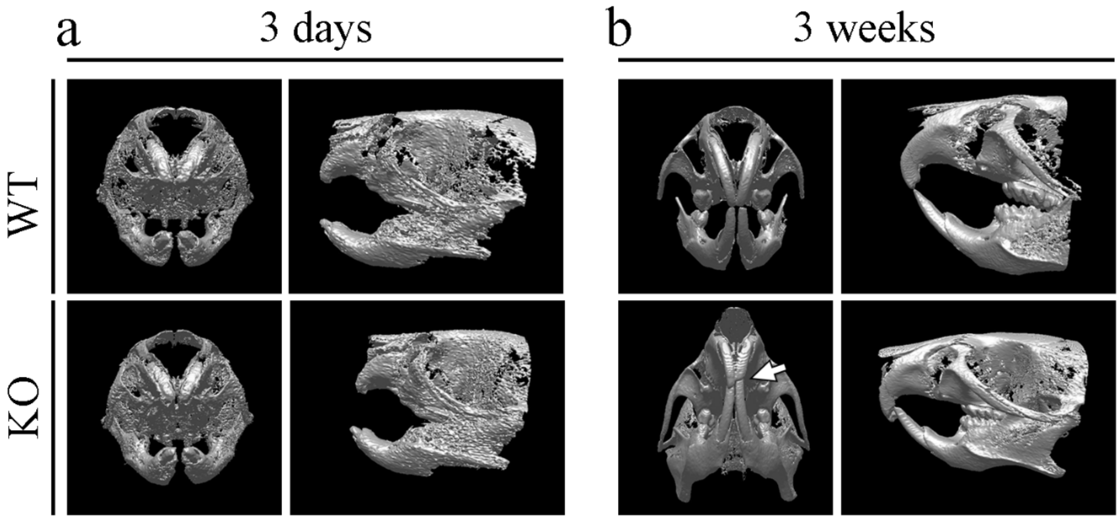

Supplementary Fig. 2. 3D reconstruction of micro-CT images of cranial skeleton at early stage.

(a) At postnatal 3 days, no differences are found between WT and *Mast4* KO cranium. (b) Asymmetrical attrition is found in *Mast4* KO incisors (arrow) compared to WT. Molars do not show morphological difference between WT and *Mast4*.

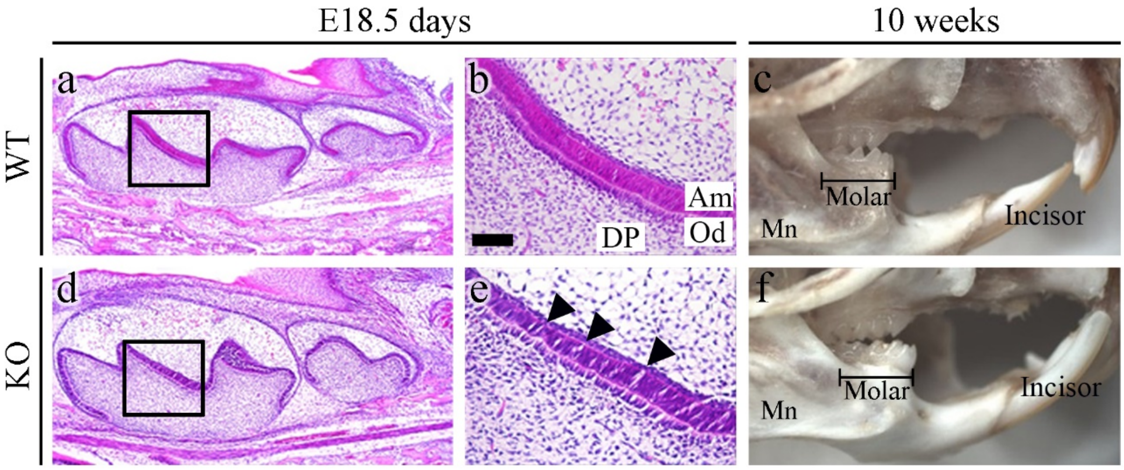

Supplementary Fig. 3. Molar development is not affected by the ablation of *Mast4*.

(a, b, d, e) Mandibular molar tooth germ of E18.5 WT and *Mast4* KO mice. Bell-stage tooth germs have elongated and well-aligned ameloblast layers. (e, arrowheads) Several defects in ameloblast alignment of *Mast4* KO tooth germ. (c, f) Mandibular bone and teeth of 10 weeks WT and *Mast4* KO mice after soft tissue removal. Contrary to the difference in length and direction of incisors, no difference was found between the WT and *Mast4* KO molars.

Am, ameloblast; DP, dental papilla; Od, odontoblast; Mn, mandible. scale bars; B, E, 200 μm.

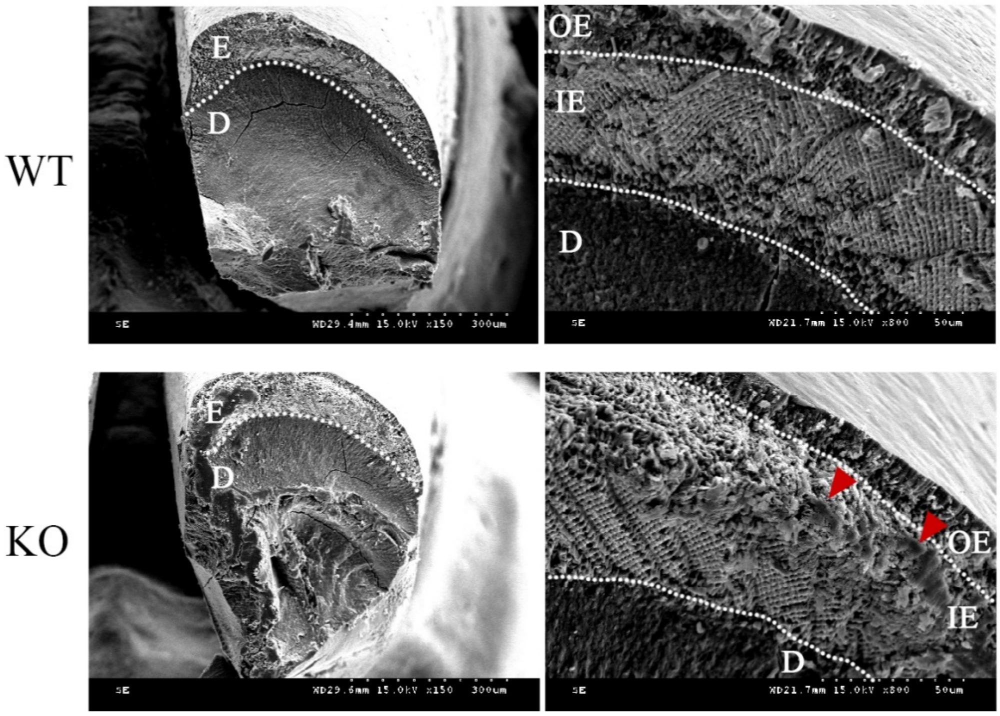

Supplementary Fig. 4. Scanning electron microscope (SEM) images of incisor dissected plane.

Outer enamel (OE) of Mast4 KO incisor is thicker than that of WT incisor. Decussated enamel rods are observed in the inner enamel (IE) layer of the WT incisor. Enamel rod arrangement in the area of OE adjacent area is collapsed in the Mast4 KO incisor (arrowheads).
D, dentin.

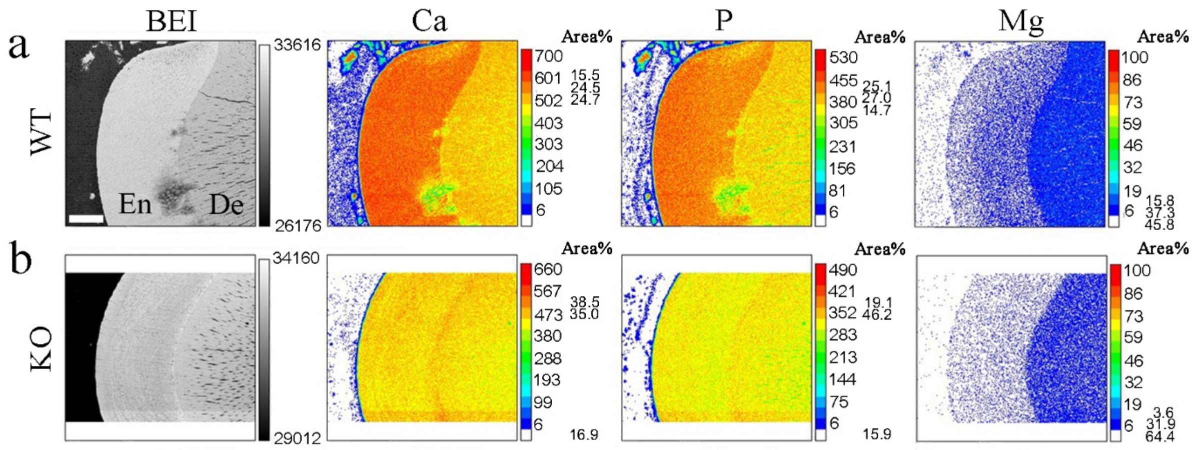

Supplementary Fig. 5. Mineral density of mandibular incisors.

(a, b) Electron probe microanalyzer (EPMA) analysis of 6-week mandibular incisors. The mineral composition of the enamel is decreased in the *Mast4* KO incisors. Numbers in addition to the indicator bars are mineral intensity (a.u.). The area % values show the three most measured intensity ranges. BEI, backscattered electron image; Ca, calcium; P, phosphorus; Mg, magnesium. Scale bars; A, B, 50 μm.

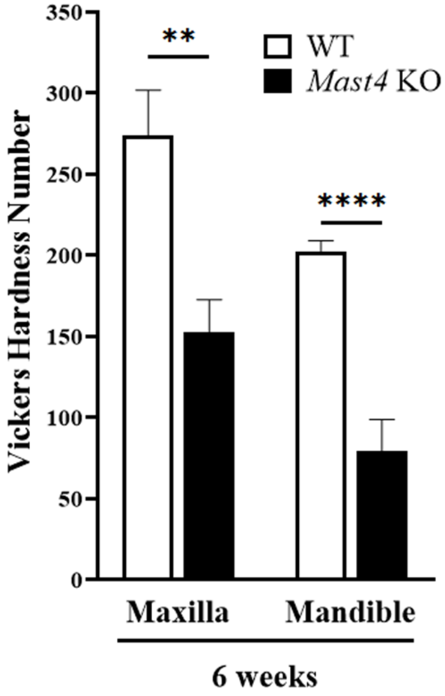

Supplementary Fig. 6. Vickers micro hardness test.

Vickers microhardness test shows that Mast4 KO incisors are weaker than WT incisors (mean ± SD, $n = 20$ per group). ** $p < 0.01$, **** $p < 0.0001$

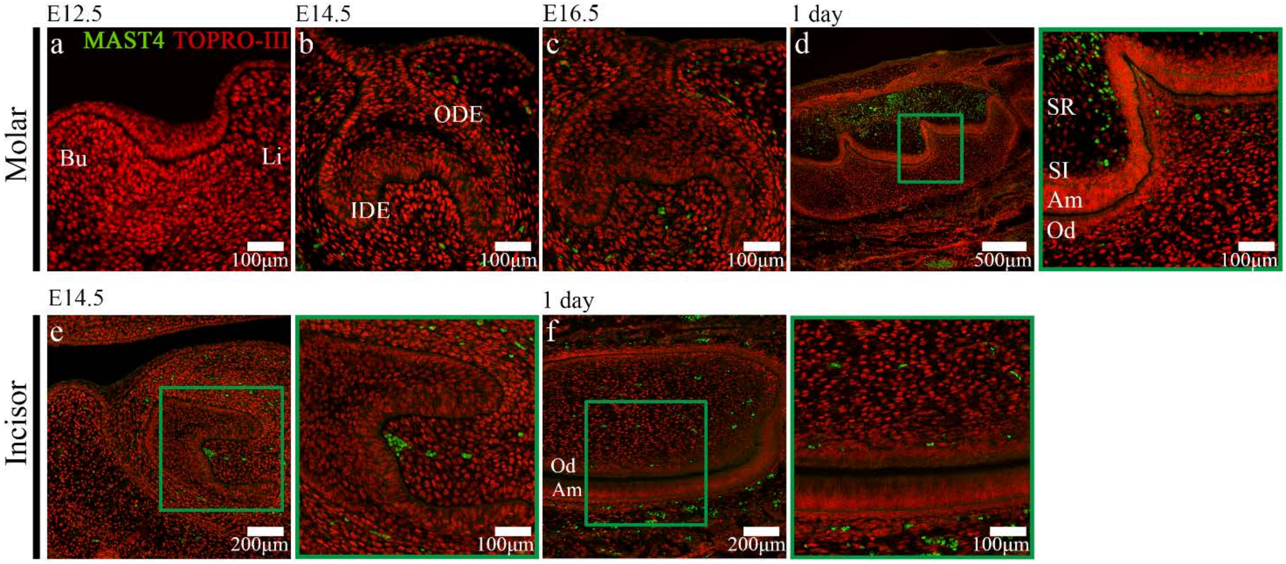

Supplementary Fig. 7. MAST4 expression during tooth development.

(a-c) MAST4 expression is not observed in molar tooth germ at E12.5, E14.5, and E16.5. Green signals detected outside the dental epithelium are due to the autofluorescence of red blood cells. (d) MAST4 is dispersed in the stellate reticulum (SR) of bell stage molar. MAST4 is not observed in stratum intermedium or ameloblast layer. Since blood vessels do not enter the dental epithelium, all signals within the SR are not from the red blood cells. Bu, Buccal; Li, Lingual; IDE, inner dental epithelium; ODE, outer dental epithelium; SR, stellate reticulum; SI, stratum intermedium; Am, ameloblast; Od, odontoblast.

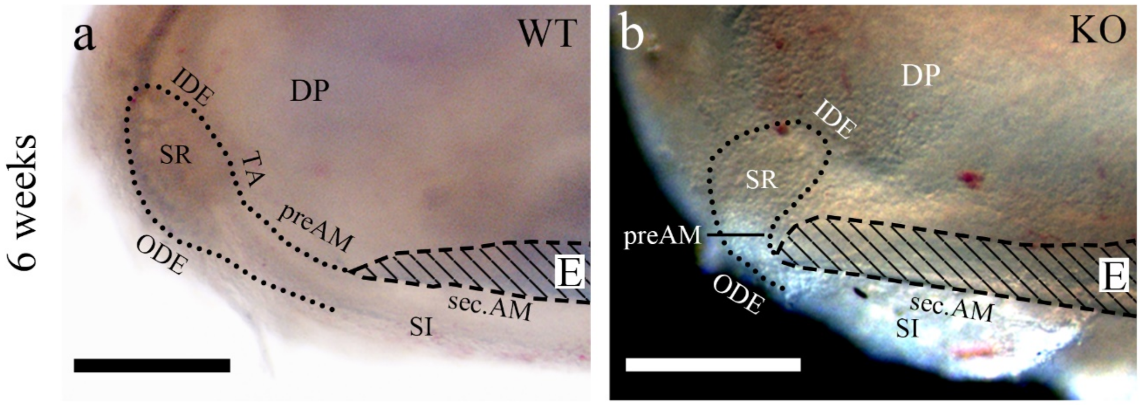

Supplementary Fig. 8. Labial cervical loops of mandibular incisors dissection.

Under a stereoscopic dissecting microscope, mandibles of 6-week WT and *Mast4* KO mice are dissected to expose the apical buds. (a) In the WT mandibular incisor, initiation of the enamel matrix was observed after the TA zone. (b) The initiation of enamel in the *Mast4* KO incisor was shifted to the apical side, and the cervical loop was reduced compared to the WT.

Dotted lines, margin of apical buds; E, enamel; DP, dental papilla; sec.Am, secretory ameloblast; preAm, Preameloblast; IDE, inner dental epithelium; ODE, outer dental epithelium; SR, stellate reticulum; SI, stratum intermedium; TA, transit-amplifying zone. All scale bars; 250 μm.

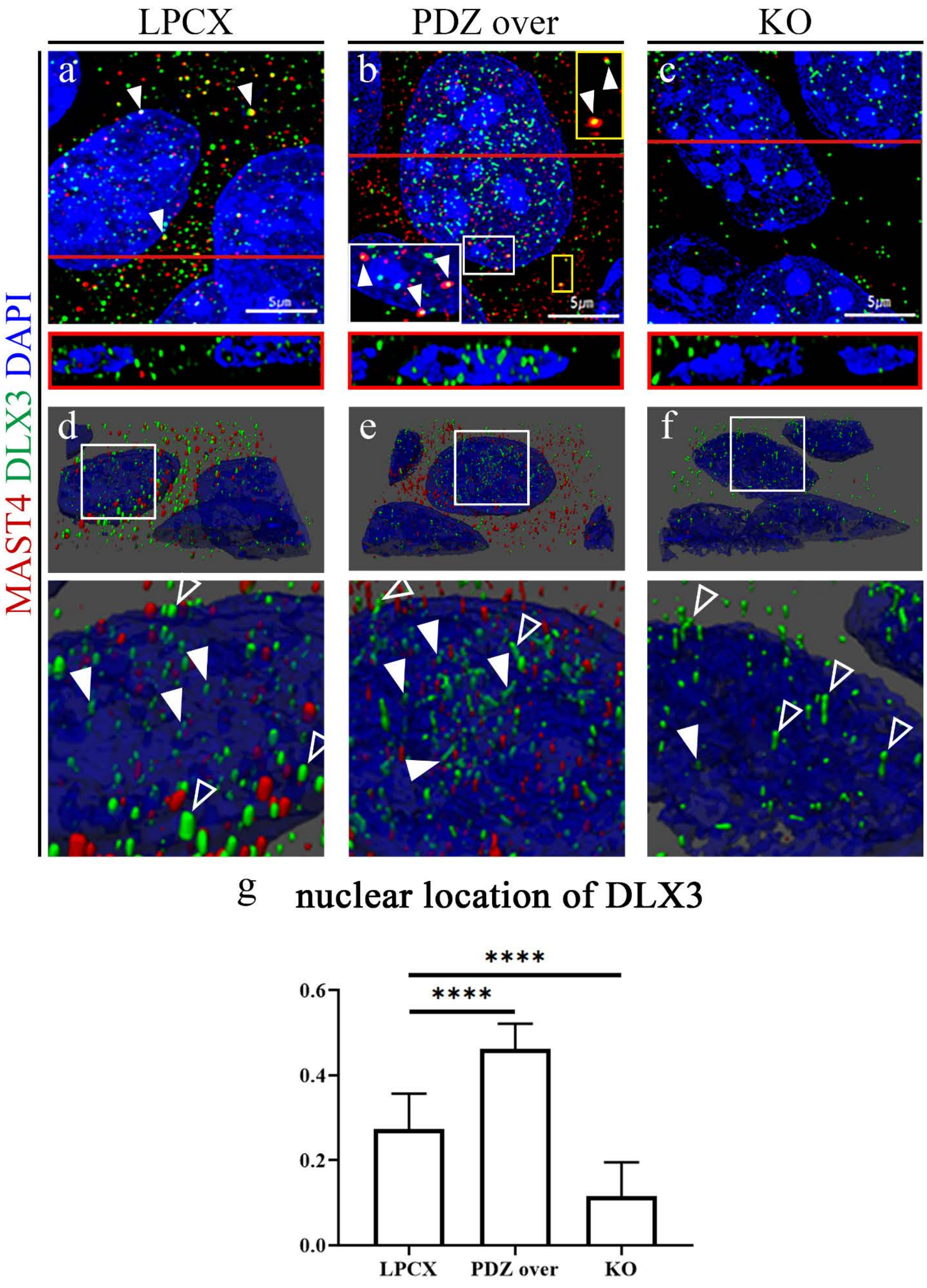
LPCX
PDZ over
KO
MAST4 DLX3 DAPI
a
b
c
d
e
f
5μm
5μm
5μm
g
nuclear location of DLX3
****
****
0.6
0.4
0.2
0.0
LPCX
PDZ over
KO

Supplementary Fig. 9. Nuclear location of DLX3 and co-localization with MAST4.

(a-c) The maximum intensity projections of z-stack images show MAST4 and DLX3 localization in mHAT9d cells. Their vertical cross-sections (red line) show nuclear localization of DLX3. Arrowheads indicate colocalization of MAST4 and DLX3. (d-f) 3D reconstruction of panel a-c. DLX3 localized both in nucleus (arrowheads) and out of nucleus (blank arrowheads). (g) Nuclear localization of DLX3 was quantified from cross-section images (mean ± SD). 20 cross-sectional images were obtained from 3 z-stack images for each group (n = 60), and the nuclear localization ratio of the DLX signal was measured. **** p<0.0001

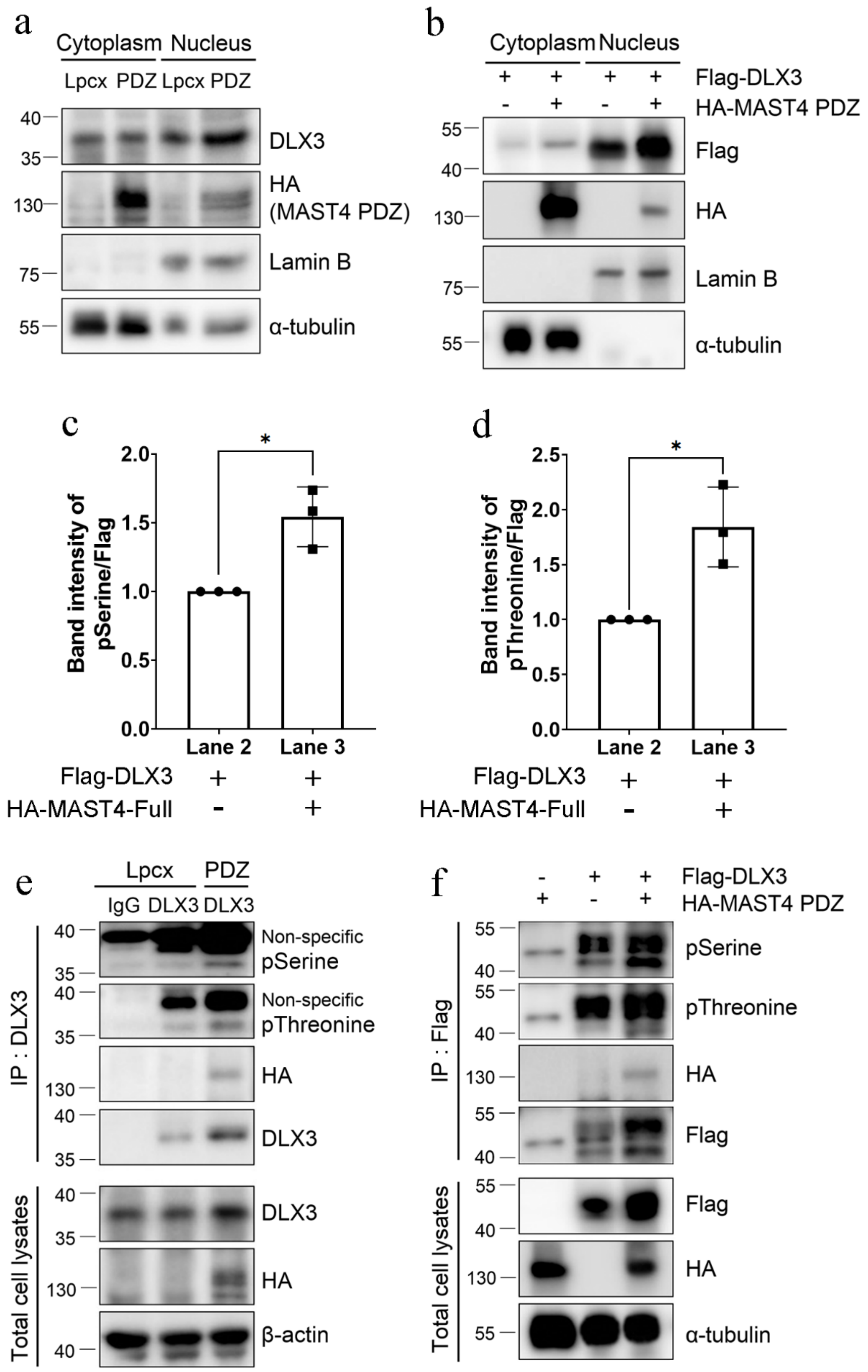

a
Cytoplasm Nucleus
Lpcx PDZ Lpcx PDZ
DLX3
HA (MAST4 PDZ)
Lamin B
α-tubulin
b
Cytoplasm Nucleus
Flag-DLX3
HA-MAST4 PDZ
Flag
HA
Lamin B
α-tubulin
c
Band intensity of pSerine/Flag
Lane 2
Lane 3
Flag-DLX3
HA-MAST4-Full
d
Band intensity of pThreonine/Flag
Lane 2
Lane 3
Flag-DLX3
HA-MAST4-Full
e
Lpcx PDZ
IgG DLX3 DLX3
IP : DLX3
Non-specific
pSerine
Non-specific
pThreonine
HA
DLX3
Total cell lysates
DLX3
HA
β-actin
f
Flag-DLX3
HA-MAST4 PDZ
IP : Flag
pSerine
pThreonine
HA
Flag
Total cell lysates
Flag
HA
α-tubulin

Supplementary Fig. 10. Nuclear location and phosphorylation of DLX3 with MAST4 PDZ overexpression.

(a, b) Analysis of DLX3 expression using subcellular fractionation in transient transfection in both PDZ domain overexpressing mHat9d stable cell line (a) and transient transfection in HEK293T cell line (b). The expression of α-tubulin in the cytoplasm and Lamin B in the nucleus served as controls for the efficiency of subcellular fractionation. (c, d) Quantification of Band intensity of pSerine and pThreonine to Flag-DLX3 in Fig. 5J. (e, f) Flag-DLX3 was immunoprecipitated, and the complexes were analyzed by western blot. Note that DLX3 phosphorylation is increased in the presence of MAST4 PDZ in mHat9d (e) and HEK 293T cell line (f). Lpcx; empty retroviral vector for control. Data are represented as mean ± SD (n = 3). *p < 0.05, **p < 0.01, ***p < 0.001, ns; non-significant.

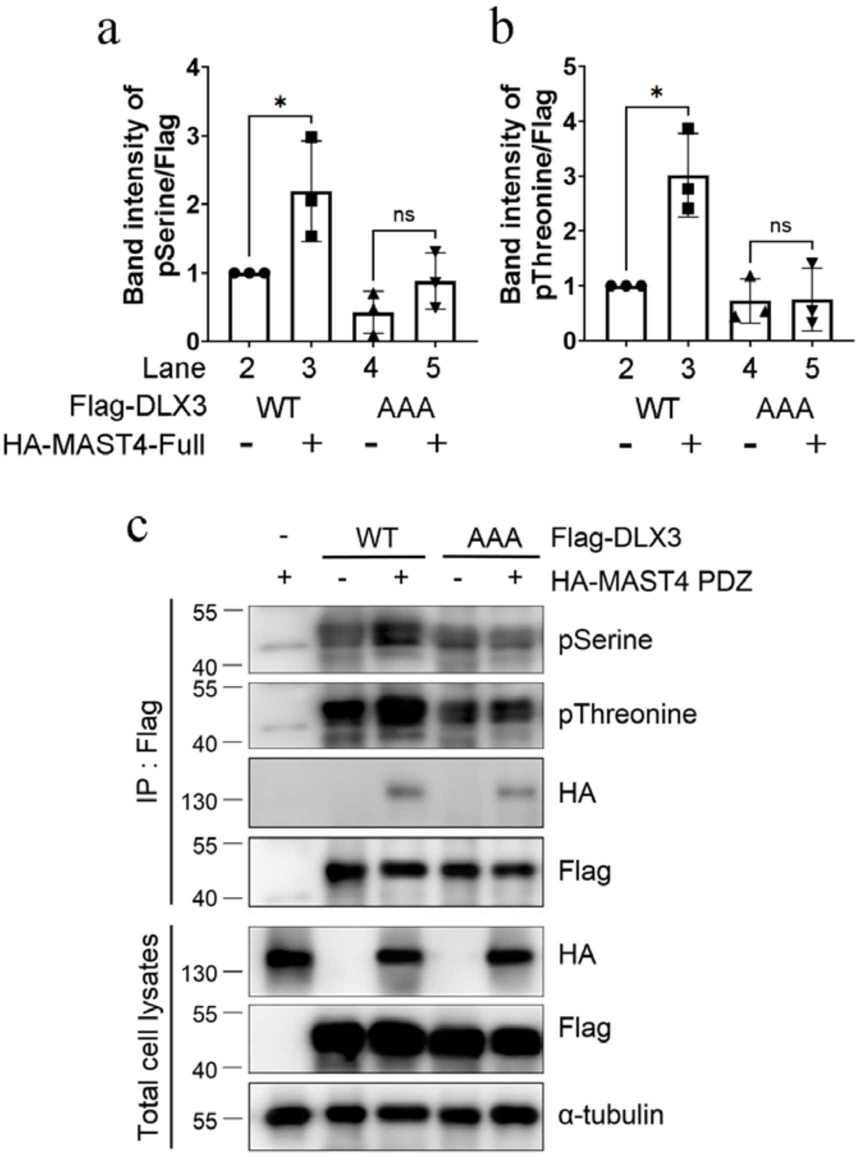

Supplementary Fig. 11. Examination of DLX NLS phosphorylation using DLX3 WT and AAA mutant by HA-MAST4-PDZ overexpression.

(a, b) Quantification of band intensity of pSerine and pThreonine to Flag-DLX3 in Fig. 5M. (c) Flag-DLX3^{WT}, DLX3^{AAA} mutant and HA-MAST4 PDZ were transiently co-transfected into HEK293T cells. Flag-DLX3 was immunoprecipitated, and the complexes were analyzed by western blot. Note that DLX3 phosphorylation is increased in the presence of MAST4. Data are represented as mean ± SD (n = 3). *$p < 0.05$, **$p < 0.01$, ***$p < 0.001$, ns; non-significant.

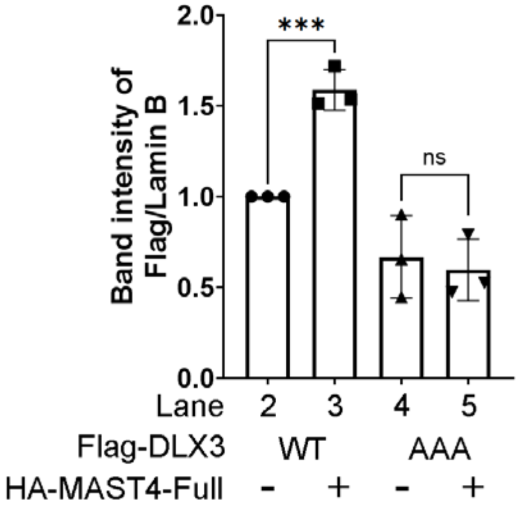

Supplementary Fig. 12. Quantification of Band intensity of Flag to Lamin B (nucleus fraction) in Fig. 5N.

Data are represented as mean ± SD (n = 3). *p < 0.05, **p < 0.01, ***p < 0.001, ns; non-significant.

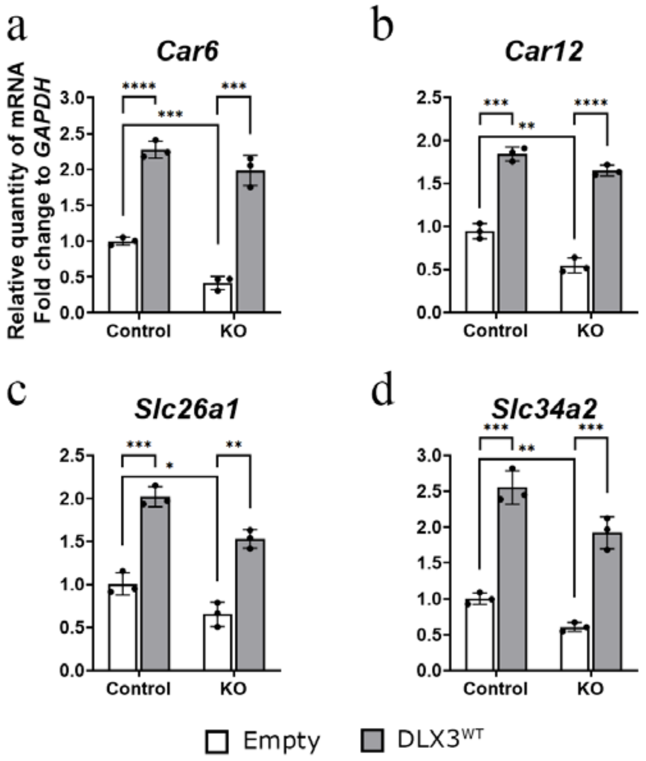

Supplementary Fig. 13. RT-qPCR results of DLX3 target genes following DLX3 WT transient transfection in *Mast4* KO mHat9d.

The expression of carbonic anhydrases (a, b) and ion transporters involved in pH regulation (c, d) was increased by DLX3 WT transiently transfection in both control and *Mast4* KO mHat9d cells. Data are represented as mean ± SD (n = 3). * $p < 0.05$, ** $p < 0.01$, *** $p < 0.01$, **** $p < 0.01$

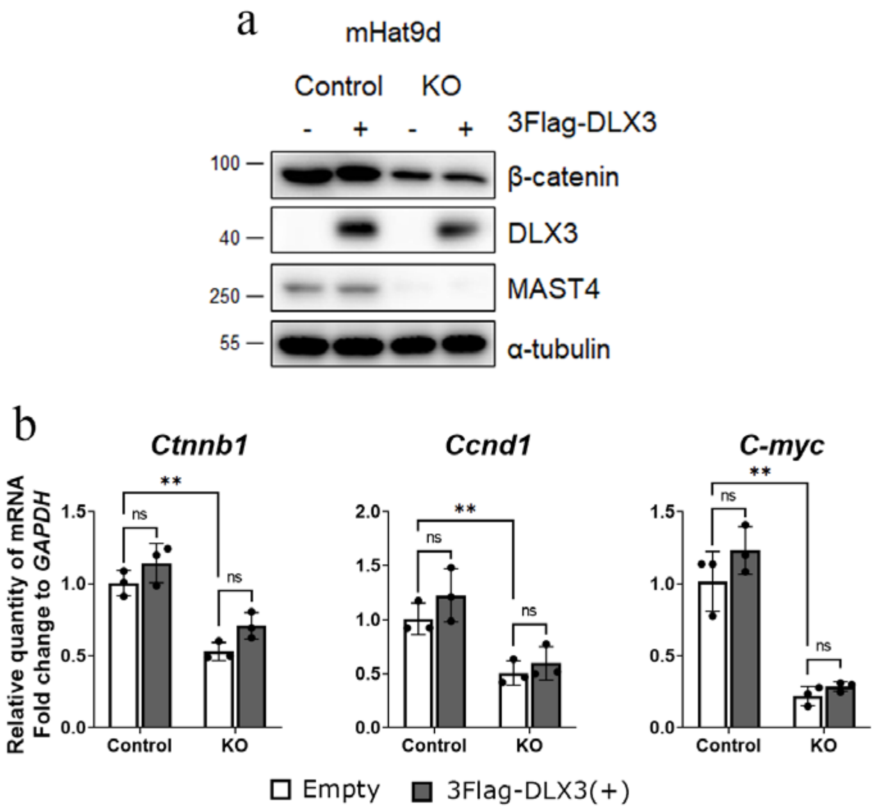

Supplementary Fig. 14. Comparative analysis of *Wnt* signaling in both control and *Mast4* KO mHat9d following Flag-DLX3 transient transfection.

(a) β-catenin expression upon transient overexpression of DLX3 in mHAT9d cells. Note that DLX3 transfection exerts no influence on *Wnt* signaling in either control or *Mast4* KO cells. (b) qRT-PCR to analyze *Wnt* signaling genes. Data are represented as mean ± SD (n = 3 for B). *p < 0.05, **p < 0.01, ns; non-significant.

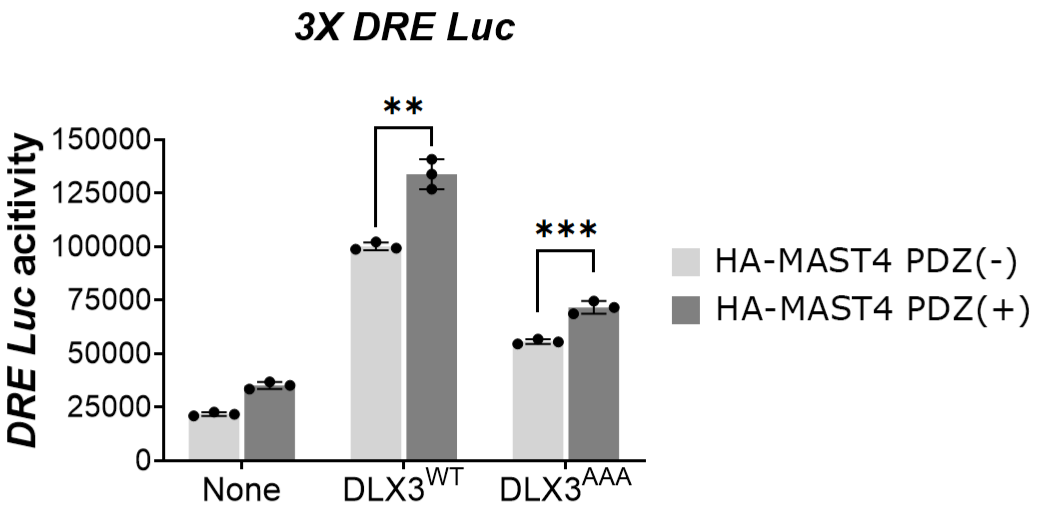

Supplementary Fig. 15. Luciferase reporter assay using 3x-DRE Luc using DLX3 WT and AAA mutant with HA-MAST4-PDZ overexpression.

3x DRE-luc, DLX3, and MAST4 PDZ were transiently overexpressed in the HEK293T cells and beta-galactosidase was co-transfected for normalization. Luciferase activities were measured after 48 h. Data are represented as mean ± SD (n = 3). **p < 0.01, ***p < 0.001, ns; non-significant.

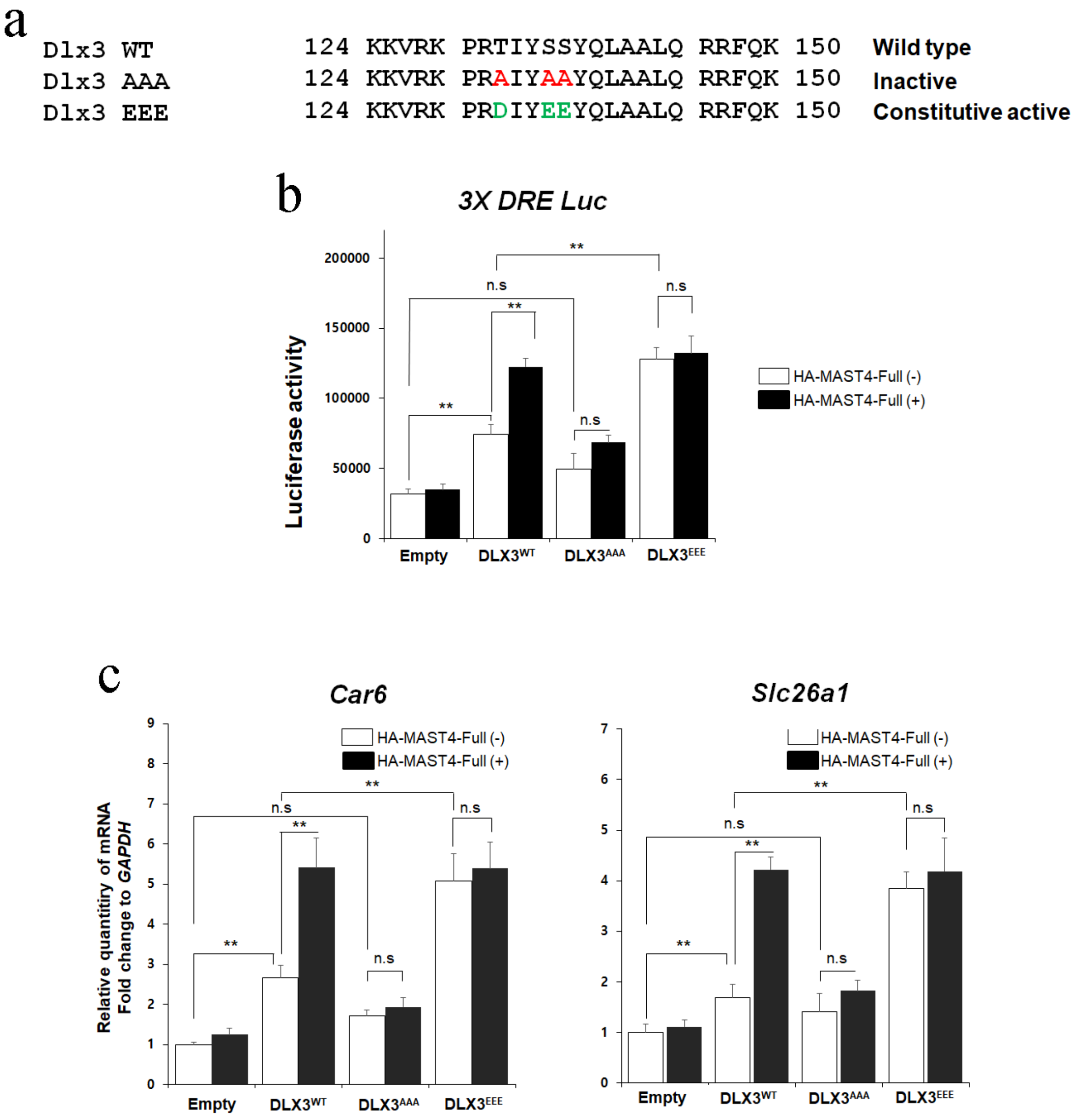

Supplementary Fig. 16. Examination of transcriptional activity of DLX3 WT, AAA and EEE mutants by HA-MAST4-Full overexpression.

(a) Sequence information of NLS phosphorylation sites mutant of DLX3. (b) 3x *DRE luc*, DLX3 WT, DLX3 AAA, DLX3 EEE and HA-MAST4-Full were transiently overexpressed in the HEK293T cells and beta-galactosidase was co-transfected for normalization. Luciferase activities were measured after 48 h. (c) RT-qPCR of carbonic anhydrases and ion transporters involved in pH regulation. DLX3 WT, DLX3 AAA, DLX3 EEE and HA-MAST4-Full were transiently overexpressed in the mHat9d cells. Data are represented as mean ± SD (n = 3 for B, n = 3 for C). *$p < 0.05$, **$p < 0.01$, ns; non-significant.

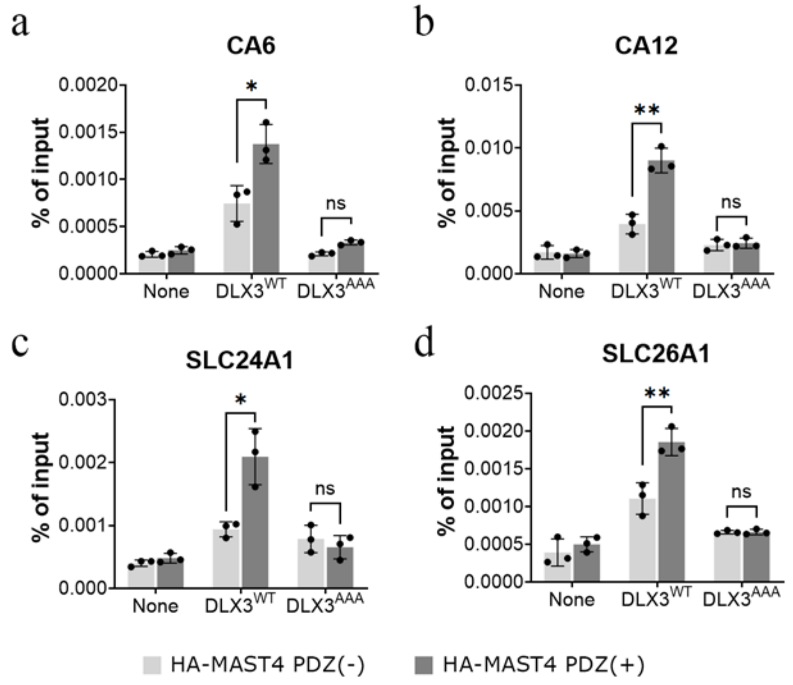

Supplementary Fig. 17. RT-qPCR of the ChIP assay of Carbonic anhydrases and Ion transporters in mHat9d cells.

$DLX3^{WT}$, $DLX3^{AAA}$ and HA-MAST4-PDZ were transiently transfected in the mHat9d cells. ChIP assay shows that $DLX3^{WT}$ increased target gene promoter binding and HA-MAST4 PDZ co-transfection further increased whereas $DLX3^{AAA}$ has no significance. (a, b) Carbonic anhydrases. (c, d) Ion transporters. Data are represented as mean ± SD (n = 3 for a-d). Data are representative of three independent experiments. * $p < 0.05$, ** $p < 0.01$, ns; non-significant.

Supplementary Table

Gene	Forward (5'-3')	Reverse (5'-3')
**Indel mutation confirm**	GTA GGG ACT CCA CGC TCC AG	CCG GAC CCT AGT CTC TTC G
Wnt-3a	GCA CCA CCG TCA GCA ACA	GGG TGG CTT TGT CCA GAA CA
Ctnnb1	GTC ATC CCC CAC ACA AAG ATG	CAC CCC TTT CCG CTC TCA
Lrp5	CCC GAG GGA GCC TTT CTA CT	CTT GCA CGT CTT GCC ATT GT
B2m	CCT GGT CTT TCT GGT GCT TG	CCG TTC TTC AGC ATT TGG AT
Ccnd1	TTG ACT GCC GAG AAG TTG TG	CCA CTT GAG CTT GTT CAC CA
C-myc	AAA GAC AGC ACC AGC CTG AG	ACT GAG GGG TCA ATG CAC TC
Car6	ACC TCT GAC GGC ACT GAG TT	CCC ATC AAT GGT GTG TTC AG
Car12	GTT CGA TGA GAG GCT GGT GT	GGG CCA CTG AAA GGA TGA TA
Cftr	CAT TCT TCA CGC CCC TAT GT	CTC CTG CCT TCA GAT TCC AG
Slc26a1	CCC ACA GGT ACC AGA CCC TA	CTG CCA AGG AGA TGG AGA AG
Slc34a2	AGC TTG TTG GAG GCA AAG TG	TAT CAC CAG TCC AGC CAC AG
Dlx3	AGC CCA GTA TCT GGC CTT G	CGG CAC CTC CCC ATT CTT A
Mast4	AGC CCA TTT TTC ATT TGC AC	TCG TCT GGT GTT GGT TGG TA
Gapdh	AAT CCC ATC ACC ATC TTC CA	TGT GGT CAT GAG TCC TTC CA

Supplementary Table 1. Primers for target site insertion and RT-qPCR.
